# Preventing HIV and achieving pregnancy among HIV sero-different couples: Pilot study of a safer conception intervention in Zimbabwe

**DOI:** 10.1371/journal.pgph.0000796

**Published:** 2023-02-24

**Authors:** Joelle M. Brown, Serah Gitome, Bismark Mataveke, Thandiwe Chirenda, Allen Matubu, Gift Chareka, Charles Chasakara, Nyaradzo Mgodi, Caroline Murombedzi, Petina Musara, Tinei Makurumure, Carolyn Smith Hughes, Elizabeth Bukusi, Craig R. Cohen, Stephen Shiboski, Lynae Darbes, James G. Kahn, George W. Rutherford, Z. Michael Chirenje, Felix Mhlanga

**Affiliations:** 1 Department of Obstetrics, Gynecology, Reproductive Sciences, University of California, San Francisco, San Francisco, California, United States of America; 2 Department of Epidemiology and Biostatistics, University of California, San Francisco, San Francisco, California, United States of America; 3 Centre for Microbiology Research, Kenya Medical Research Institute, Nairobi, Kenya; 4 University of Zimbabwe, Harare, Zimbabwe; 5 University of Zimbabwe Clinical Trials Research Centre, Harare, Zimbabwe; 6 Mercy-Care Fertility Centre, Harare, Zimbabwe; 7 Department of Health Behavior and Biological Sciences, University of Michigan, Ann Arbor, Michigan, United States of America; McGill University, CANADA

## Abstract

Safer conception services are needed to minimize HIV transmission among HIV sero-different couples desiring pregnancy. Few studies have evaluated the choices couples make when offered multiple safer conception methods or real-world method acceptability and effectiveness. We piloted a comprehensive safer conception program (*Clintrials*.*gov* identifier: NCT03049176) for HIV sero-different couples planning pregnancy in Zimbabwe to measure feasibility, method uptake, acceptability, pregnancy outcome, and HIV transmission. This study was not designed to compare rates of HIV transmission by safer conception method choice but rather to understand choices couples make when seeking to minimize risk of HIV transmission and maximize likelihood of pregnancy. Couples in this prospective, non-randomized study were given a choice of one or more currently available safer conception methods: antiretroviral therapy (ART) with monthly viral load (VL) monitoring for the HIV-positive partner (ART/VL), pre-exposure prophylaxis (PrEP) for the HIV-negative partner, vaginal insemination (VI) for couples with an HIV-positive woman, and semen washing (SW) for couples with an HIV-positive man. Couples were followed monthly for up to 12 months of pregnancy attempts, quarterly during pregnancy, and 12 weeks post-partum. At each visit, data on method use, urine for pregnancy testing, and blood for HIV antibody testing, or viral load if HIV-positive, were obtained. Infants born to HIV-positive women were tested for HIV at 6 and 12 weeks. Between March 2017 and June 2019, 46 individuals from 23 HIV sero-different partnerships were enrolled and followed. At enrollment, all couples chose ART/VL, and all couples chose at least one additional method; 74% chose PrEP, 36% chose SW, and 25% chose VI. During pre-pregnancy follow-up visits, three couples discontinued SW, and one couple discontinued VI; all four of these couples opted for ART/VL plus PrEP. Satisfaction with safer conception methods was high among those who chose ART/VL and PrEP. Twelve couples achieved pregnancy. There were no cases of HIV transmission to partners, and no infants tested positive for HIV. This safer conception program is feasible and acceptable, allowing sero-different couples to safely achieve pregnancy. Sero-different couples in Zimbabwe seek a combination of HIV prevention methods, particularly ART/VL plus PrEP.

**Trial Registration:** Clintrials.gov, NCT03049176.

## Introduction

Across sub-Saharan Africa (SSA), the vast majority of people living with HIV are of reproductive age [1]. With the scale up of antiretroviral therapy (ART), HIV-positive individuals are living longer, healthier lives, and both the desire to conceive and pregnancy rates have increased [2, 3]. It is estimated that among HIV-positive people who are in stable relationships, up to one half include an HIV-negative partner [4, 5]. If HIV viral suppression is not achieved or sustained, these HIV sero-different couples (one partner is HIV-positive while the other is not) are at risk for HIV transmission to the HIV-negative partner [6, 7], particularly when having vaginal intercourse without a condom in order to achieve pregnancy [8–10].

Multiple HIV-prevention methods are available and can be used to reduce HIV transmission risk during pregnancy attempts [11], including ART with viral load suppression for the HIV-positive partner [12–14], pre-exposure prophylaxis (PrEP) for the HIV-negative partner [15, 16], semen washing (SW) for couples in which the male partners are HIV-positive [17], and vaginal insemination (VI) for couples in which the female partners are HIV-positive [18, 19]. Numerous studies have demonstrated the effectiveness of ART and PrEP on reducing HIV transmission risk, and that these methods are more effective when taken consistently [20, 21]; ART with viral load suppression is increasingly being linked with no sexual HIV transmission [12–14] while daily oral PrEP has been found to be 92% effective among women whose drug levels suggest daily use of PrEP [15, 22]. Semen washing [17] and VI [18, 19] are estimated to reduce HIV transmission risk by 100%. The World Health Organization (WHO) recommends ART for all people living with HIV and PrEP for discordant couples; however, there are no global safer conception guidelines and few countries in HIV endemic settings have country-level policies [11, 23–27], leaving many providers unprepared to counsel the vast majority of women and men in need of safer conception services [28–30].

A systematic review of the availability of safer conception services in SSA found that while individual safer conception methods (e.g. ART, PrEP, VI, SW) are available in a number of settings, they are generally not provided in the context of safer conception programs and the implementation of safer conception services has largely been limited to research projects [23]. For example, while ART has been scaled up in SSA, it is not generally provided in the context of safer conception counselling and services and viral load suppression may not be routinely assessed [31]. Access to PrEP is increasing, though its availability continues to remain limited in many settings throughout SSA [32, 33]. The availability of SW is limited due to expense, though efforts to make SW more accessible and lower cost are underway [34]. VI, while low-cost, is infrequently discussed with patients outside of safer conception research projects [23, 35].

To date, most of the research on safer conception services in SSA has been qualitative, hypothetical, and cross-sectional and has found that couples would accept various safer conception methods if offered but that knowledge of safer conception among patients and providers is limited [23, 36, 37]. Data from prospective safer conception studies are needed, particularly in settings where HIV is endemic, to understand method preferences, evaluate the feasibility and potential impact of safer conception services, and optimize service delivery and guidelines.

We conducted a prospective pilot study of a comprehensive safer conception program among a cohort of HIV sero-different couples attempting pregnancy in Zimbabwe, a country that has been hit particularly hard by the HIV pandemic with 1.2 million reproductive age adults living with HIV and an estimated 25,000 new HIV infections per year [1]. The goals of this non-randomized pilot study were to measure the feasibility of implementing the program, and the uptake, use, and acceptability of available safer conception methods, as well as the effectiveness of the program in allowing sero-different couples to achieve pregnancy and avoid HIV transmission to HIV-negative partners and infants. This study was not designed to compare rates of HIV transmission by safer conception method choice but rather to understand choices couples make when seeking to minimize risk of HIV transmission and maximize likelihood of pregnancy. This is the first study of its kind in Zimbabwe and may inform the delivery of safer conception services among HIV sero-different couples in Zimbabwe and similar settings.

## Materials and methods

### Study overview and site

This study, known as the SAFER study (*Clintrials*.*gov* identifier: NCT03049176), was conducted from March 2017 to June 2019 within the University of Zimbabwe Clinical Trials Research Centre (UZ-CTRC), a collaborative research program between the University of California, San Francisco (UCSF) and the University of Zimbabwe. The SAFER study was conducted at the UZ-CTRC Zengeza clinical research site located on the grounds of the Zengeza Municipality Clinic in Chitungwiza, Zimbabwe, which provides primary care, maternity care, family planning, and well-baby services.

To build community awareness and foster preparedness for the SAFER study, we engaged existing UZ-CTRC Community Advisory Board members comprising local stakeholders, such as people living with HIV, youth, local health providers, and local leaders. We worked with HIV counseling and testing centers and opportunistic infection treatment clinics in Harare and Chitungwiza to refer sero-different couples to the Zengeza site for screening and possible enrollment in the study.

### Study design and procedures

**Fig 1:**
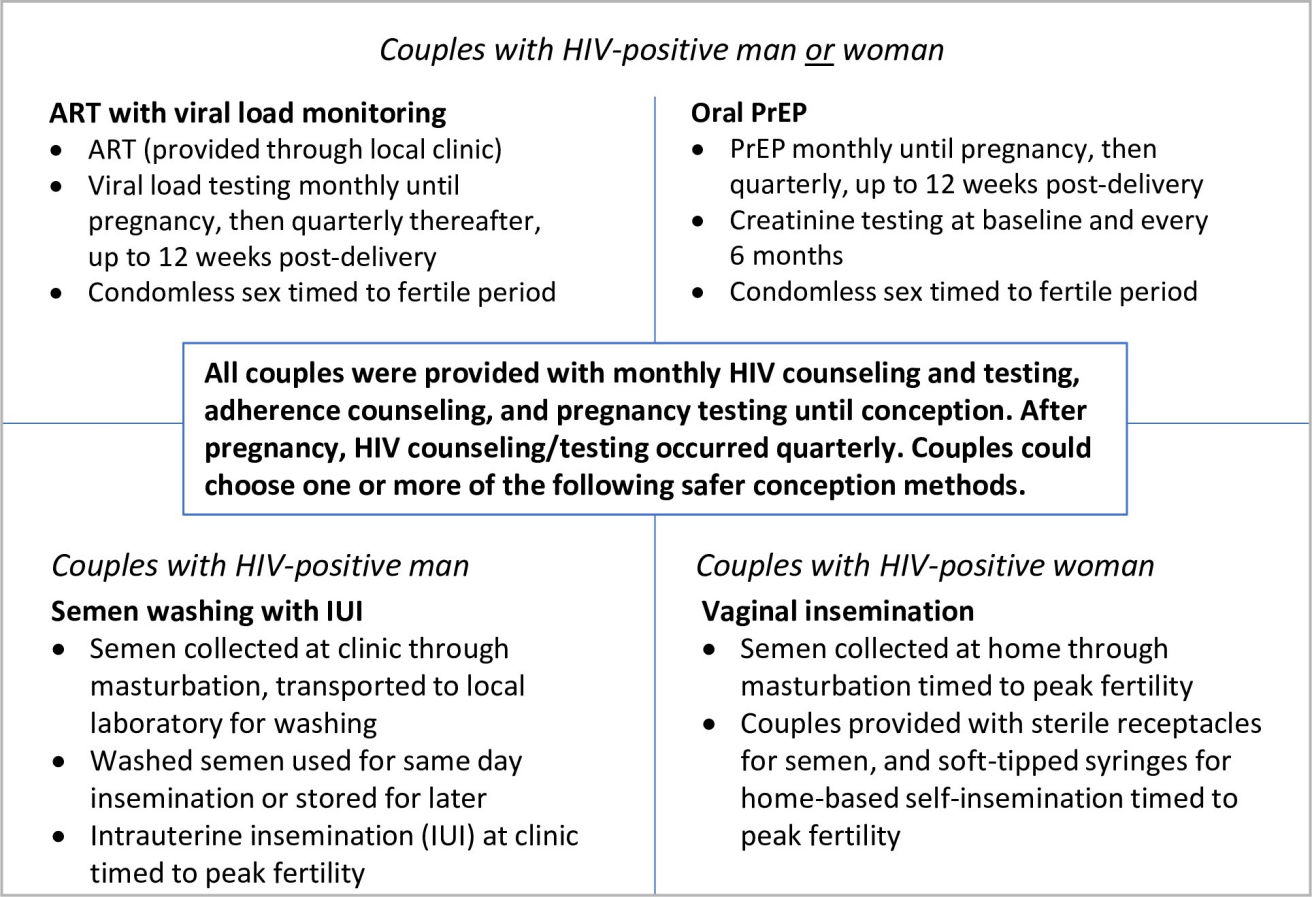
Safer conception methods offered in the study.

This was a prospective, non-blinded, non-randomized pilot study of a comprehensive safer conception program provided to all enrolled couples. We enrolled a cohort of HIV sero-different couples in Zimbabwe hoping to achieve pregnancy within the next 6 months. All couples were counseled on the use of safer conception methods available in Zimbabwe (Fig 1) and allowed to choose one or more methods: ART with monthly viral load monitoring (ART/VL) for the HIV-positive partner, PrEP with daily oral tenofovir disoproxil fumarate (200mg)/emtricitabine (300mg) (TDF-FTC) for the HIV-negative partner, home-based VI at the time of ovulation for couples with an HIV-positive woman, and SW with intrauterine insemination at the time of ovulation for couples with an HIV-positive man. While we considered a randomized design, safer conception methods were not randomly allocated in this study given ethical considerations, which precluded denying the intervention to some, and logistical, financial and timeline constraints, which did not allow us to follow multiple cohorts for long periods of time (e.g. stepped-wedge design). Each couple was counselled on how to track the female partner’s menstrual cycle and identify her days of peak fertility and provided with paper calendars for the purpose of charting menses and predicting fertile periods. All couples were provided with nutritional counseling, and women were given folic acid supplementation as per the national guidelines.

Prior to conception attempts, all enrolled couples underwent a two-month run-in period during which they returned to the clinic each month to review menstrual cycle calendars and receive additional counseling on tracking, as needed, as well as HIV counseling and testing for HIV-negative partners, VL testing for the HIV-positive partners, and pregnancy testing for women. Participants who opted for PrEP as a safer conception method were initiated on PrEP during the run-in phase to ensure PrEP was taken at least 20 days before pregnancy attempts began [38].

**Fig 2:**
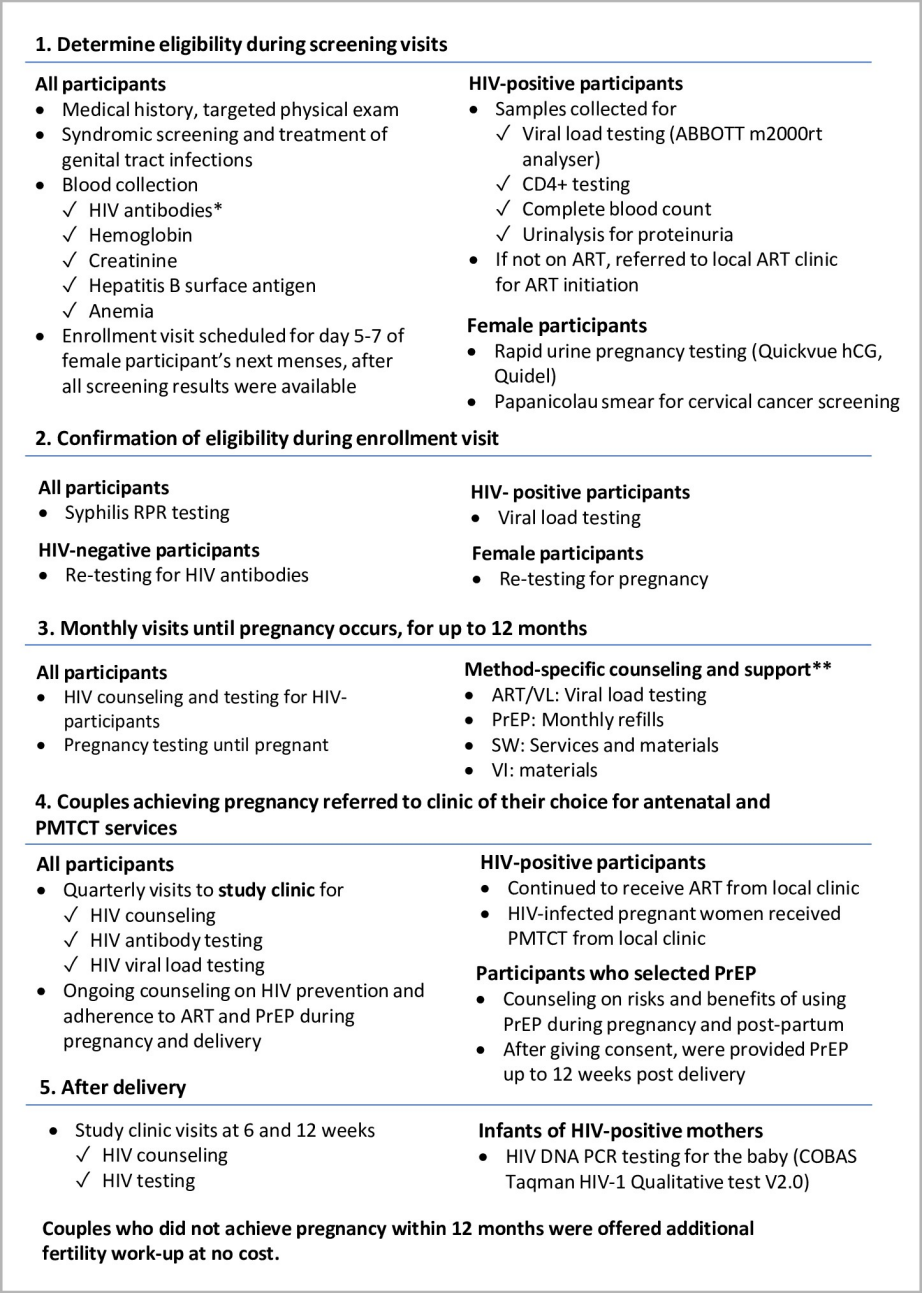
SAFER study procedures and clinical and laboratory work-up. *Using 3rd generation HIV rapid antibody tests, Determine HIV-1/2 and Oraquick, with confirmation of positives using 4th Generation BioRad GS HIV 1/2 COMBO EIA. **See Fig 1 for additional details on each of the safer methods.

Upon completion of the run-in period, couples were counselled to begin conception attempts with their chosen safer conception method(s) and to return to the study clinic monthly for up to 12 months for urine pregnancy testing, HIV counselling and testing, HIV VL testing, and targeted counselling on adherence to their selected safer conception methods, guided by an adherence counselling toolkit specifically developed for healthcare providers offering safer conception to HIV sero-different couples [39]. Couples were counselled to always use condoms, except for those using PrEP or ART/VL who had condomless sex during the peak fertile period. Male condoms were offered at every visit at no cost. Couples were allowed to switch safer conception methods during follow-up. Once pregnancy occurred, couples returned to the study clinic for quarterly visits, and at 6- and 12-weeks following delivery for HIV testing. Couples were referred to their local clinic of choice for continuation of ART, antenatal care (ANC), and prevention-of-mother-to-child-transmission (PMTCT) services. Participants who selected PrEP as safer conception were counselled on the risks and benefits of taking PrEP during pregnancy and post-partum. After giving consent, they were provided with PrEP for up to 12 weeks post-delivery (Fig 2).

### Study population and procedures

**Fig 3:**
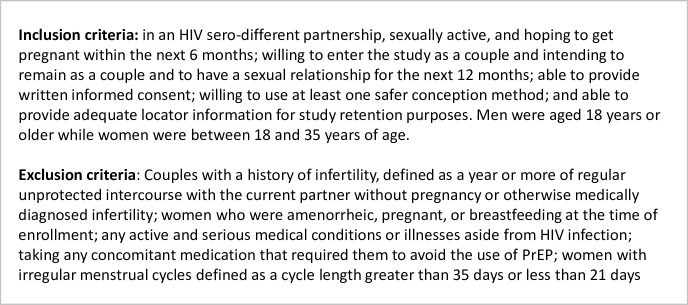
SAFER inclusion and exclusion criteria.

We enrolled HIV sero-different couples in Zimbabwe who were hoping to get pregnant within the next 6 months. Eligibility determination and clinical and laboratory procedures are described in detail in Figs 2 and 3, and in the study protocol (S1 Text). Laboratory testing was conducted at the UZ-CTRC central laboratory in Harare according to national guidelines. HIV-positive participants who were not on ART at screening were referred to the local ART clinic of their choice for ART initiation.

**Fig 4:**
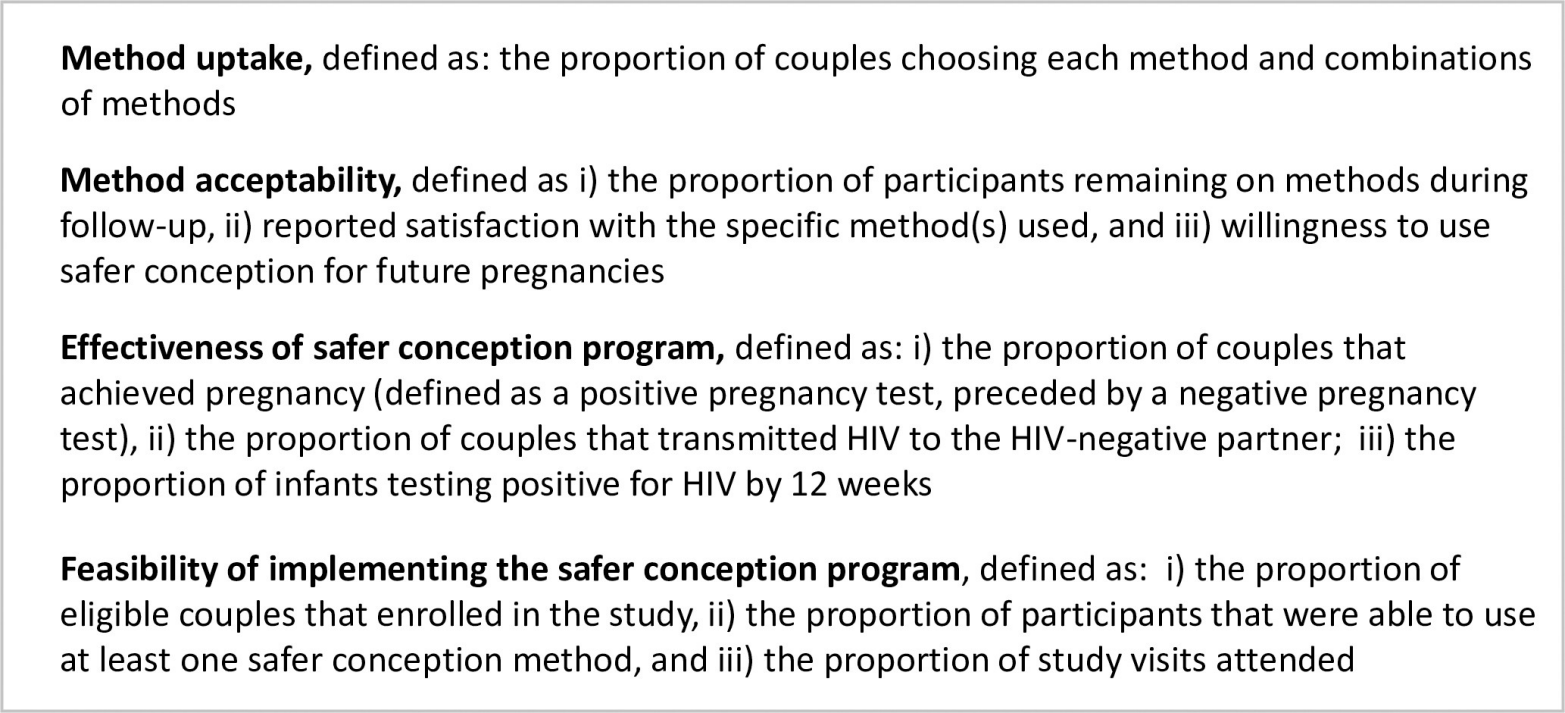
Definitions of outcome measures.

Primary outcomes for this analysis were safer conception method uptake, method acceptability, effectiveness of the safer conception program (proportion of women who achieve pregnancy, incident HIV infection in HIV-negative partner and baby), and feasibility of implementing the safer conception program, as defined in Fig 4.

### Data management and analysis

**Fig 5:**
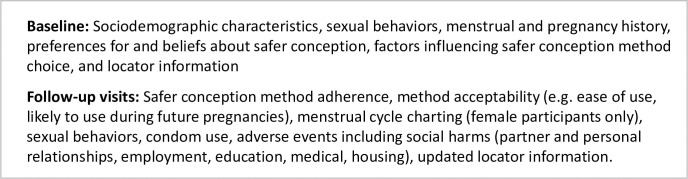
Sociodemographic and behavioral data collected from all couples at baseline and during follow-up.

Study data were collected and managed using REDCap (Research Electronic Data Capture; RedCAP Consortium, Vanderbilt University, Nashville, Tennessee, USA) electronic data capture tools hosted at UCSF. Sociodemographic and behavioral data (Fig 5) were collected using tablet-based structured questionnaires, which were administered verbally and in-person by study staff with each member of the couple separately and concurrently at baseline and follow-up visits and transmitted in real-time over a secure cellular data network to UCSF. Formal power calculations were not undertaken as this was designed to be a small pilot study, and the sample size was determined by available funding. As a result, this pilot study was not powered to compare rates of HIV transmission by safer conception method choice. We performed descriptive statistics using STATA, v 16 (STATA Corporation, College Station, Texas, USA) to summarize socio-demographic characteristics; uptake of, preferences for, adherence to, and acceptability of safer conception methods; pregnancy; and HIV outcomes. *P-values* were not reported due to the small sample size of this pilot.

### Ethical considerations for human subject research

The study protocol and all participant-related materials were approved by the University of California, San Francisco Institutional Review Board, the Municipality of Chitungwiza, the Joint Research Ethics Committee of the University of Zimbabwe, the Medical Research Council of Zimbabwe, the Medicines Control Authority of Zimbabwe and the Research Council of Zimbabwe. Participant materials were translated into the local language (Shona). Each couple member was consented individually in his or her preferred language (English or Shona) to minimize coercion, and provided written informed consent. Participants experiencing social harms were counselled on-site and referred for further management as necessary.

## Results

**Fig 6:**
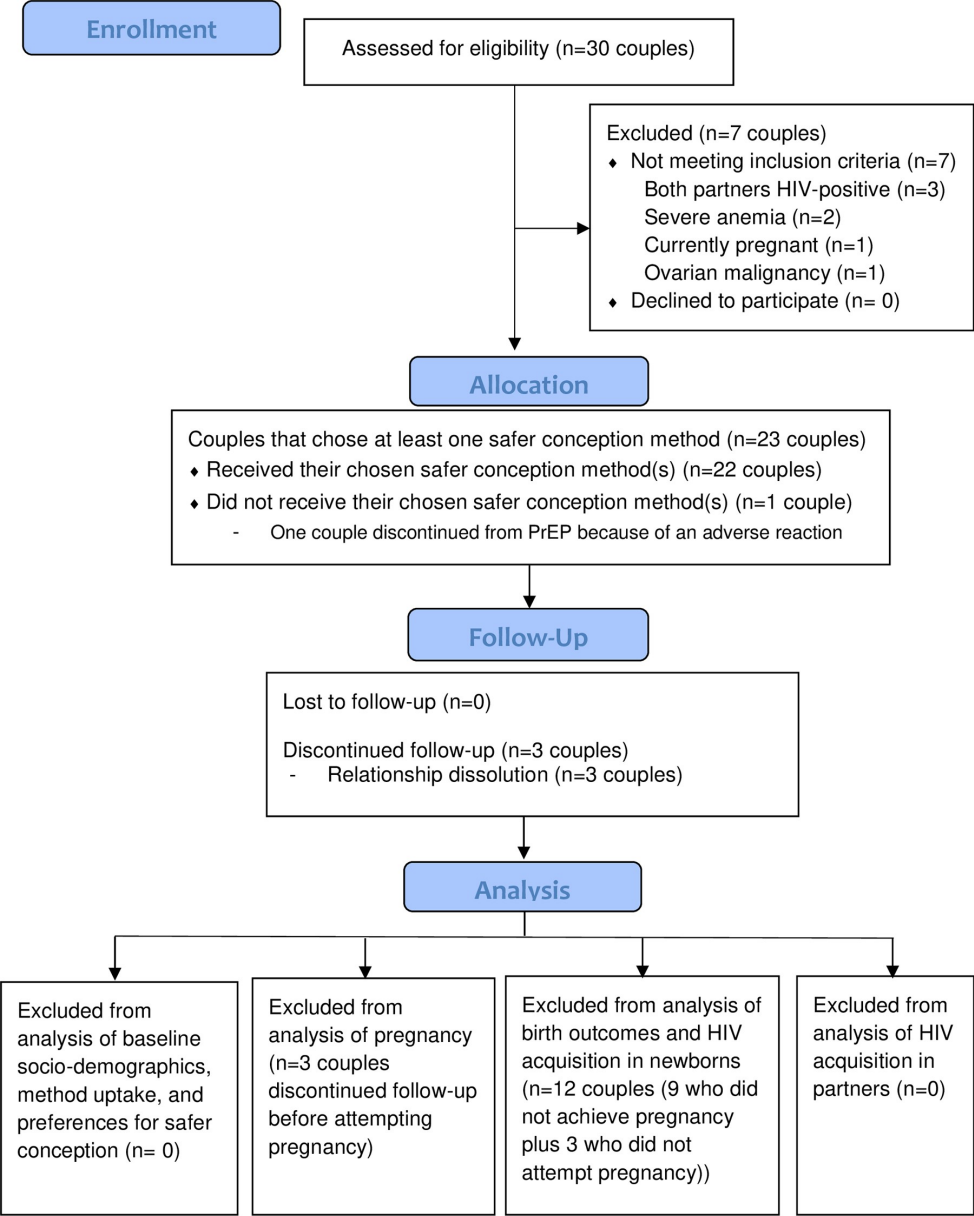
Enrollment and follow-up of participants.

Of the 30 couples screened, seven were ineligible for the following reasons: both partners HIV-positive (n = 3), severe anemia (n = 2), current pregnancy (n = 1), and ovarian malignancy (n = 1) (Fig 6). All 23 eligible couples (46 participants) enrolled into the study. Half of the couples (12/23, 52·2%) had an HIV-positive female partner. No couples were lost to follow-up; however, prior to attempting pregnancy three couples (3/23, 13%) withdrew participation due to relationship dissolution. Nearly all enrolled couples returned for each scheduled monthly and quarterly follow-up visit per protocol; 91% (497/549) of possible study visits were attended, and all HIV-negative participants had HIV testing conducted at study exit.

### Baseline characteristics

The median age for women was 31 years (interquartile range (IQR): 27–34; range: 21–35) and 34 years for men (IQR: 32–41; range: 24–54) (Table 1). At enrollment, all HIV-positive participants were on ART, with a median baseline CD4 count of 673 cells/mm3 (IQR: 334–864). Five (21·7%) HIV-positive participants were not virally suppressed (viral load ≥40 copies/mL) at enrollment, and two of these participants continued to have detectable viral load at the end of the two-month run-in period (viral load: 19,000 and 34,000 copies/mL) despite reports of high adherence. These two participants had drug resistance testing, were switched to second-line therapy, and asked to delay pregnancy attempts until suppression was achieved (time to viral suppression was 4 and 6 months, respectively).

**Table 1:** Baseline characteristics of enrolled couples.

	Men	Women
	(n = 23)	(n = 23)
	Median (IQR) or n (%)	
Age (years)	34 (32–41)	31 (27–34)
Completed secondary education	16 (69·6)	12 (52·2)
Religious denomination		
Protestant	11 (47·8)	13 (56·5)
Catholic	1 (4·4)	2 (8·7)
Apostolic	5 (21·7)	8 (34·8)
None	6 (26·1)	0
Married to and living with study partner	23 (100)	23 (100)
Committed to maintaining relationship with current partner	23 (100)	23 (100)
Employed	20 (87·0)	11 (47·8)
Income (USD per month)	300 (120–600)	100 (30–200)
House has electricity	13 (56·5)	
House has more than one room	17 (73·9)	
HIV-positive	11 (47·8)	12 (52·2)
Length of time on ART (months) if HIV-positive	36 (8–70)	15 (3–33)
Undetectable viral load (<40 copies/mL) if HIV-positive	10/11 (90·9)	8/12 (66·7)
CD4 count (cells/mm^3^) if HIV positive	583 (312–755)	719 (493–874)
Parity	--	2 (0–3)
One or more living children with current partner	12 (52·2)	
Number of living children with current partner	1 (0–3)	
Number of living children, total	2 (0–5)	2 (0–4)
“The thought of my child contracting HIV scares me” ^a^	15 (65·2)	18 (78·3)
“The thought of contracting HIV scares me” if HIV-negative^a^	6/12 (50·0)	7/11 (63·6)

^a^ Dichotomized from 5-point Likert scale: strongly agree/agree vs neutral/disagree/strongly disagree

IQR = Interquartile range

### Method uptake and preferences at enrollment

**Fig 7:**
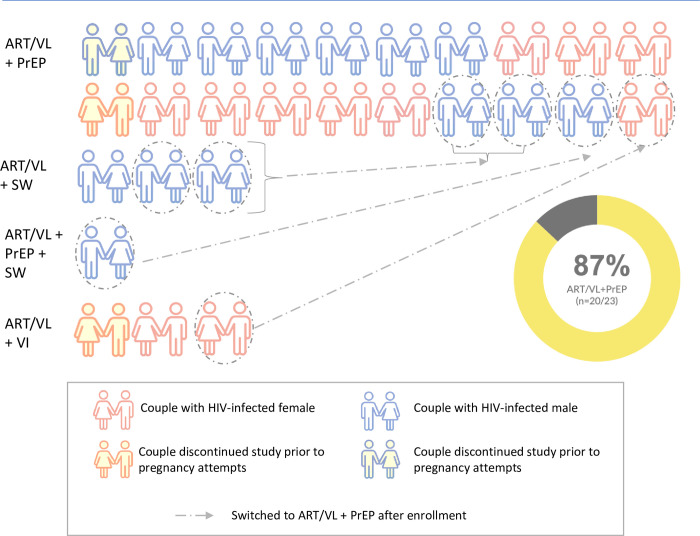
Safer conception method uptake at enrollment and switching during follow-up. ART/VL = antiretroviral therapy (ART) with monthly viral load (VL) monitoring; PrEP = pre-exposure prophylaxis, VI = vaginal insemination; SW = semen washing.

At enrollment, all couples chose at least two safer conception methods (Fig 7); all (100%) couples chose ART/VL, and all couples chose at least one additional method. PrEP was selected by 17 couples (73·9% 95%CI: 51·6–89·8%); in eight of these couples the woman was HIV-negative (Table 2). Three (25·0%) of 12 couples with an HIV-positive woman chose VI. Four (36.4%) of 11 couples with an HIV-positive man chose SW. One couple with an HIV-positive man chose to use three methods for safer conception: ART/VL, PrEP, and SW.

**Table 2:** Preferences for safer conception methods at enrolment.

	Men	Women
	n (%)	n (%)
*Among all participants*	**(n = 23)**	**(n = 23)**
**Which of the following safer conception methods would you prefer?** ^a^		
A method I have control over	16 (69·6)	9 (39·1)
A method my partner has control over	7 (30·4)	14 (60·9)
Best protection from HIV	11 (47·8)	15 (65·2)
Easiest to use	9 (39·1)	10 (43·5)
Most likely to result in pregnancy	2 (8·7)	1 (4·4)
*Among HIV-negative participants*	**(n = 12 HIV-)**	**(n = 11 HIV-)**
**I chose PrEP**	9 (75·0)	8 (72·7)
**I did not choose PrEP because:** ^a^		
Too difficult to remember to take daily pill	2 (16·7)	1 (9·1)
Burden to take daily pill	2 (16·7)	1 (9·1)
Worried about side effects	2 (16·7)	2 (18·2)
If I take daily pill, people might think I am sick	2 (16·7)	3 (27·3)
*Among couples with an HIV-positive woman*	**(n = 12 HIV-)**	**(n = 12 HIV+)**
**I chose vaginal insemination**	3 (25·0)	3 (25·0)
**I did not choose vaginal insemination because:** ^a^		
Unnatural	2 (16·7)	1 (8·3)
Too difficult	6 (50·0)	7 (58·3)
Other reason	1 (8·3)	1 (8·3)
*Among couples with an HIV-positive man*	**(n = 11 HIV+)**	**(n = 11 HIV-)**
**I chose semen washing**	4 (36·4)	4 (36·4)
**I did not choose semen washing because:** ^a^		
Unnatural	1 (9·1)	0 (0)
Too difficult	4 (36·4)	5 (45·5)
Other reason	2 (18·2)	2 (18·2)

^a^ Multiple responses allowed

At enrollment, approximately two-thirds (15/23, 65·2%) of women and half of men (11/23, 47·8%) reported choosing a particular method because they “believed it offered the best protection against HIV” (Table 2). Couples that selected ART/VL and PrEP reported doing so because these methods are “easiest to use” and “most likely to protect us from HIV than the other methods” (Table 3).

**Table 3:** Reasons ^a^ that couples (N = 23) gave for selecting their chosen safer conception method(s) at enrolment.

	ART/VL	PrEP	VI	SW/IUI
	n/N (%)			
More likely to protect us from HIV than other methods	13/23 (56·5)	9/17 (52·9)	2/3 (66·7)	3/4 (75·0)
Easier to use than other methods	10/23 (43·5)	9/17 (52·9)	1/3 (33·3)	0/4 (0)
More likely to result in pregnancy than other methods	1/23 (4·4)	1/17 (5·9)	0/3 (0)	2/4 (50·0)

^a^ Multiple responses allowed

### Method switching and self-reported adherence

Four couples chose to switch safer conception methods during follow-up (Fig 7); two couples using ART/VL+SW discontinued SW and began using ART/VL+PrEP, one couple using ART/VL+PrEP+SW discontinued SW and continued using ART/VL+PrEP, and one couple using ART/VL+VI discontinued VI and began using ART/VL+PrEP. Overall, 20 (86·9%, 95%CI: 66·4–97·2%) of the 23 enrolled couples chose to use ART/VL+PrEP for safer conception in this study. One participant on PrEP permanently discontinued PrEP due to an adverse reaction (see Adverse Events below).

Self-reported ART and PrEP use was high throughout the study. At enrollment, the median number of reported ART doses over the past 30 days was 30 (IQR: 30–30; range: 29–30), and no participants reported >4 missed ART doses per month at any point during study follow-up. During the first month of pregnancy attempts, the median number of missed doses of PrEP was 0 (IQR: 0–0) and most (14/17, 82·4%) participants reported taking ≥4 doses of PrEP per week while trying to conceive. Reported rates of ART and PrEP adherence were similar for men and women.

### Pregnancy and birth outcomes

Twenty (86·9%) of the 23 enrolled couples attempted pregnancy. The three couples that did not attempt pregnancy withdrew participation due to relationship dissolution; two of these couples were using ART/VL+PREP, and one was using ART/VL+VI. Nearly two-thirds of couples achieved pregnancy within the year; 12 (60·0%, 95%CI: 36·1–80·9%) women (6 HIV-positive, 6 HIV-negative) achieved 14 pregnancies. There were 11 live births (nine singletons and one set of twins), with a median birthweight of 2600 grams (IQR: 2530–2800), no congenital anomalies, 3 first-term miscarriages, and 1 stillbirth. All women reported tracking the fertile period during the first month of pregnancy attempts. During study follow-up, women reported spending a median of 20 minutes (IQR: 5–30) per month tracking their fertile period.

The median age of women who became pregnant was 31 years (IQR: 28–33) (one of whom was nulliparous), and male partners’ median age was 34 years (IQR: 32–36). The median time to pregnancy was 2 months (range 1–9). Among couples who achieved pregnancy, 11 pregnancies occurred using ART/VL+PrEP, one while using ART/VL+VI and two while using ART/VL+SW. Nine (90·0%, 95%CI: 55·5–99·7%) of 10 participants using PrEP chose to continue PrEP during the pregnancy and post-partum period (5 of 5 women and 4 of 5 men). Eight couples did not get pregnant despite trying for a median of 7 months (IQR: 4–10). The median age of women who did not get pregnant was 32 years (IQR: 27–34) (none were nulliparous), and their male partners’ median age was 41 (IQR: 36–44). None of these couples opted to undergo a fertility evaluation.

All three miscarriages occurred in the first trimester of pregnancy and were among HIV-negative women. Two of these women were taking PrEP, one couple was using SW, and all three male partners were on ART and virally suppressed. One stillbirth occurred at 38 weeks gestation to a 32-year-old primiparous, HIV-positive woman who had been taking ART for the previous 21 months. Throughout study participation and pregnancy this participant had an undetectable viral load; a CD4 count that ranged from 845–861 cells/mm^3^; no evidence of anemia; no pre-eclampsia (blood pressure consistently under 130/90), no proteinuria or glycosuria; no significant weight loss; took iron and folic acid supplementation; no trauma; normal fetal growth as measured by fundal height; no per vaginal bleeding or ulcers; no signs or symptoms of sexually transmitted infections and tested negative for syphilis; normal fetal heart rate; and attended ANC visits as recommended. Local antenatal health card data suggest a medically uneventful pregnancy up to the two days, at 38 weeks, she could not perceive fetal movements and experienced mild labour contractions. The following day, the participant progressed with the stages of labour and delivered a macerated stillborn fetus (2700 grams) with normal gross phenotype. The clinical history and abrupt loss of fetal movements in this case suggest the most likely cause to be concealed placental hemorrhage. These adverse event rates are comparable to the rates of miscarriage among HIV-negative women of 25–35% [40] and a frequency of stillbirth among HIV-positive women on ART of 6% [37, 41, 42].

### HIV transmission

There were no cases of sexual HIV transmission during the pre-pregnancy, pregnancy, and post-partum follow-up periods. Once virally suppressed, 21 (91·3%) of the 23 HIV-positive participants maintained a viral load of <40 copies/mL at study follow-up visits. For the two HIV-positive participants with detectable viral load during follow-up (one man and one woman), values ranged from 52–180 copies/mL. None of the 11 infants tested positive for HIV nucleic acid by 12 weeks post-partum.

### Adverse events

One participant (1/46; 2·2%, 95%CI: 0·1–11·5%) experienced an adverse event (AE) considered related to one of the study interventions. This woman was taking PrEP (1/20; 5·0%, 95%CI: 0·1–24·9%) and experienced a Grade 2 generalized erythematous, pruritic rash one day after initiating PrEP. This was associated with headache, dizziness, and fever and resulted in permanent discontinuation of PrEP. None of the participants taking PrEP had serum creatinine elevations. Six participants experienced a total of six other serious adverse events (SAE): hospitalization to rule out typhoid fever, pelvic inflammatory disease, three first-term miscarriages, and one stillbirth. None of the SAEs were considered possibly, probably or definitely related to study interventions (S2 Text). All AEs and SAEs resolved by the end of the study. Five women reported social harm during the study related to their male partner: all five women reported quarrelling with their partner, one of these women reported mild physical violence by her partner, and none reported the harm to be related to study participation. Three of these couples separated during the study and discontinued study follow-up prior to pregnancy attempts.

### Method acceptability

Satisfaction with safer conception methods was high among those who chose ART/VL and those who chose PrEP. At study exit, all 19 couples that used ART/VL+PrEP for at least one month said both ART/VL and PrEP are “very easy methods to use for safer conception”, and all couples said they are “likely or very likely to use ART/VL” and “likely or very likely to use PrEP” for future pregnancies. Of the two couples that used VI during conception attempts, one couple said it is a “very easy method to use for safer conception” and they are “likely or very likely to use vaginal insemination” for their future pregnancies. The one couple that discontinued VI did so after 2 months of using this method and stated that it was “too complicated and difficult to put semen in syringe and inseminate”. Only one of four couples that used SW said they are “likely or very likely to use SW for future pregnancies” and that it is “an easy method to use”. The three couples that discontinued SW did so after 2, 3 and 5 months of using this method, respectively and gave several reasons for discontinuation including it was “difficult to provide a semen sample”, (n = 2) “too complicated” (n = 1), “have concerns about paternity” (n = 1) and “desire condomless sex” (n = 1).

## Discussion

The SAFER pilot study found that offering a comprehensive package of safer conception services is feasible and achieved excellent results; we observed high uptake of safer conception methods, high satisfaction with and adherence to ART and PrEP, excellent retention among men and women, nearly two-thirds of couples achieving pregnancy—a pregnancy rate comparable to similar settings [36, 43]—and no cases of HIV transmission to HIV-negative partners or newborns. Our results align with two recently published safer conception feasibility studies in Kenya [36] and South Africa [37]. The Kenyan study offered ART and PrEP to 74 HIV sero-different couples and found that both are acceptable as safer conception methods and observed no cases of sexual HIV transmission. The South African study included 162 HIV sero-different couples and found that the majority selected ART (91%), followed by VI (62%), and PrEP (27%); approximately half of couples achieved pregnancy within 12 months, and no HIV seroconversions were observed.

The SAFER pilot study suggests that couples prefer to use a combination of safer conception methods, even when the HIV-positive partner is taking ART, with ART/VL plus PrEP being the most commonly selected combination. Both the Kenyan and South African studies found that many couples opt for a combination of safer conception methods; however, the methods chosen varied between the different settings. Providing safer conception counselling and permitting couples to select their preferred method may lead to increased uptake [44], as well as adherence, as has been found in the contraceptive literature [45]. Allowing sero-different couples to choose a combination of prevention methods may also be important given their relatively high risk of HIV transmission during pregnancy attempts, and our observation that not any one prevention method was used 100% of the time. For example, not all participants on ART were virally suppressed at enrollment, some had detectable HIV viral loads during pre-pregnancy follow-up visits, and not all participants took all doses of ART or PrEP. We know from numerous studies that ART and PrEP are most effective when taken consistently [20, 21], especially for women [46]. While undetectable viral load is increasingly being linked with no sexual HIV transmission, in some settings it may not be feasible to monitor viral load to a point where ART use alone can successfully be used to prevent HIV transmission among HIV sero-different couples attempting pregnancy. As such, our participants’ desires to use a combination of prevention methods are welcome and may have contributed to their success in preventing HIV transmission [47].

Given that women’s HIV acquisition risk more than doubles during pregnancy and the postpartum period compared to when women are not pregnant [48, 49], safer conception programs may want to ensure that PrEP is available throughout pregnancy and breastfeeding to protect the health of HIV-negative women and their infants, an approach supported by World Health Organization recommendations [50]. To date, few implementation studies have evaluated the real-world uptake and acceptability of this approach in settings with a high burden of HIV [51]. However, our pilot study suggests this approach is acceptable; all HIV-negative women in our study opted to continue using PrEP through pregnancy and 12 weeks post-partum. Long-acting injectable antiretroviral formulations for PrEP [52, 53] as well as for HIV treatment, that result in improved adherence and better efficacy than the oral formulations may be particularly useful for men and women in safer conception programs in the future.

Based on the findings of this pilot study, we have several recommendations for the implementation of future safer conception programs in less-resourced areas: Prioritize keeping the HIV-positive partner on ART and virally suppressed, with VL monitoring at enrollment, and if possible, during follow-up; offer PrEP as a complementary prevention method before, during, and after pregnancy; and teach couples to identify their peak fertile period in order to limit exposure to condomless intercourse. For those unable or unwilling to use PrEP or to have intercourse without a condom, programs can offer VI, and if resources permit, SW. However, both VI and SW had lower acceptability in our setting and were challenging for some couples to use. We are currently analysing in-depth qualitative interviews collected from couples and providers in the SAFER study to better understand experiences with and challenges using the methods. We summarize the costs of each of these methods in a separate publication [54], and our team is currently conducting a cost-effectiveness analysis of these safer conception methods used alone and in combination.

### Limitations and strengths

This pilot study has several limitations. Our study was limited by a relatively small sample size and a single study site which reduces the generalizability of our results and our ability to find statistical differences. Our intervention offered services to the couple and promoted shared decision-making and options based on the preferences of the couple as a whole, versus tailoring our intervention to individuals. We recognize that safer conception services may need to be expanded to address the needs of individual women and men who want to safely have children but cannot or do not wish to receive services as a couple. Safer conception services will be most beneficial to couples with planned pregnancies, which are estimated to be about half of all pregnancies among women in SSA [55]. Given that ART and PrEP are now standards of care throughout the world, we did not design this pilot as a randomized controlled trial to determine the effectiveness of one safer conception method versus another but rather as a pilot study to assess the feasibility of the overall safer conception program, the methods that couples choose to use, method acceptability and the proportion of enrolled couples that safely achieve pregnancy.

Our safer conception program has several unique strengths. We allowed couples to choose from multiple safer conception methods, including relatively novel methods such as SW and VI, and allowed couples to switch safer conception methods during follow-up. This approach enabled us to understand which method(s) the participants want to try, and which one(s) they are actually able to use. We followed couples through pregnancy and the post-partum period to ensure that partners remained HIV-negative during the high-risk pregnancy period and that babies born to HIV-positive women were also HIV-negative. We included measures of preferences for and satisfaction with methods selected, which can aid in future implementation and development of safer conception programming. We sensitized communities, trained providers and integrated our safer conception services into standards of care; services were in addition to the HIV treatment and prevention services the couples were already receiving, which was efficient and did not divert patients from their usual care.

## Conclusions and implications

Safer conception services are feasible and acceptable and allow HIV sero-different couples to safely achieve pregnancy. Safer conception guidelines and services are largely unavailable in SSA, though providers and patients express a desire to learn more about them. When offered a choice, sero-different couples desiring pregnancy often seek a combination of HIV prevention methods, though preferences vary across settings. Data from large, prospective safer conception studies in various settings and populations (e.g. couples and individuals [56]) are needed, particularly where HIV is endemic, to further describe method preferences and use, measure potential impact and cost-effectiveness, optimize service delivery and inform the development of national and global safer conception guidelines. Sensitization of communities, target populations and providers will be critical elements of future safer conception programs.

## Supporting information

S1 Checklist STROBE checklist. (DOC)

S2 Checklist CONSORT pilot feasibility checklist. (DOC)

S1 Text Study protocol. (DOCX)

S2 Text Serious adverse event listing. (DOCX)
