# Supplementary material for: Preventing HIV and achieving pregnancy among HIV sero-different couples: Pilot study of a safer conception intervention in Zimbabwe
Source: PLOS Glob Public Health. 2023 Feb 24;3(2):e0000796. doi: 10.1371/journal.pgph.0000796 (PMC10022125; doi:10.1371/journal.pgph.0000796)
Supplement: S1 Text — (DOCX) [file pgph.0000796.s003.docx]

**Study Protocol**

SAFER: The impact, feasibility, acceptability, and cost-effectiveness of safer conception strategies for HIV-discordant couples in Zimbabwe

**Version 4.0**

**12 January 2017**

**TABLE OF CONTENTS**

page

LIST OF ABBREVIATIONS iv

PROTOCOL SUMMARY vi

INVESTIGATORS AND INSTITUTIONAL AFFILIATIONS vii

1 Abstract/summary 1

2 INTRODUCTION: BACKGROUND INFORMATION AND SCIENTIFIC RATIONALE 2

*2.1* Background Information 2

*2.2* Safer conception strategies 2

*2.3* Justification 3

3 objectives 5

*3.1* Study Hypothesis 5

*3.2* General Objectives 5

*3.3* Specific Objectives and Study Outcome Measures 5

*3.4* Sample size considerations 5

4 design and methodolOgy 7

*4.1* Study Site Background 7

*4.2* Study Design 7

*4.3* Study Participants 8

*4.4* Description of Safer Conception Strategies to be offered 9

5 eligibility, ENROLLMENT AND PROCEDURES for NON-RANDOMIZED INTERVENTIONAL Pilot study (Objective 1) 14

*5.1* Inclusion and Exclusion Criteria 14

*5.2* Strategies for Recruitment and Retention 15

*5.3* Screening visit 15

*5.4* Enrollment visit and 2-month run-in period 16

*5.5* Monthly Follow-up Visits before conception occurs 18

*5.6* Quarterly Follow-up Visits after conception occurs 21

*5.7* Interim Visits 22

*5.8* Visit Windows 22

*5.9* Study Withdrawal 22

*5.10* Laboratory Procedures/Evaluations 22

6 PREP MEDICATION 24

7 assessment of safetY 25

*7.1* Data and Safety Monitoring Plan 25

*7.2* Safety Monitoring Committee 25

*7.3* SMC Process 25

*7.4* Reports 25

8 Eligibility, Enrollment and Procedures for Qualitative interviews (Objective 2) 27

*8.1* Overview 27

*8.2* Eligibility Criteria 27

*8.3* Procedures 27

9 procedures for Cost-effectiveness Study (objective 3) 29

*9.1* Overview 29

*9.2* Procedures 29

9.2.1 Micro-costing study 29

9.2.2 Time and motion studies 29

10 DATA Handling and Record keeping 31

*10.1* Data Collection 31

*10.2* Data Storage 31

*10.3* Data Management 31

*10.4* Types of Data 32

*10.5* Study Records Retention 32

Research records will be maintained for at least 3 years following the completion of the research and finalization of publications. 32

11 Statistical considerations 33

*11.1* *Analysis Plan* 33

12 Ethical considerations 35

*12.1* Ethical Considerations 35

*12.2* Institutional Review Board 35

*12.3* Process of Obtaining Informed Consent and Documentation of Consent 35

*12.4* Participant Confidentiality 36

*12.5* Potential Benefits, Risks and Plans for Mitigating Those Risks 36

12.5.1 Potential Benefits 36

12.5.2 Potential Risks and Plans for Mitigating Those Risks 37

12.5.3 Alternatives to Participation 39

12.5.4 Provisions for the Protection of Privacy 40

12.5.5 Measures for Ensuring Confidentiality of Data (access to data, duration and location of storage of data) 40

12.5.6 Plans for Disseminating Study Findings to Study Participants 40

13 Quality assurance 41

14 expected application of results 42

15 Literature References 43

APPENDIX A: Schedule of Events - Screening, Enrollment/Run-In, and Delivery of Strategies 47

Appendix B. Schedule of Events in Pregnancy and Postpartum 51

APPENDIX C: Timeline 53

LIST OF ABBREVIATIONS

AE Adverse Events

ART Antiretroviral Therapy

ALT Alanine Transaminase

AST Aspartate Transaminase

AZT Zidovudine

CBC Complete Blood Count

CD4 Cluster Designation 4

CHR Committee on Human Research

CHW Community Health Workers

CLIA Clinical Laboratory Improvement Amendments

CO Clinical Officers

DAIDS Division of AIDS

DALY Disability Adjusted Life Year

DBS Dried Blood Spot

DMPA Depot Medroxyprogesterone Acetate

DNA Deoxyribonucleic acid

EFV Efavirenz

ELISA Enzyme-Linked Immunosorbent Assay

ERC Ethical Review Committee

FANC Focused Antenatal Care

GCP Good Clinical Practice

GLP Good Laboratory Practice

HBV Hepatitis B Virus

HCP Health Care Providers

HIV Human Immunodeficiency Virus

IATA International Air Transport Association

IDI In-Depth Interview

IUI Intrauterine insemination

IRB Institutional Review Board

JREC Joint Research Ethics Committee

LPV Lopinavir

MCAZ Medicines Control Authority of Zimbabwe

MRCZ Medical Research Council of Zimbabwe

MO Medical Officers

MoH Ministry of Health

MOHCC The AIDS and TB Directorate, Ministry of Health and Child Care (Zimbabwe)

MTCT Mother to Child Transmission

NIH National Institutes of Health

NMTPAC National Medicine and Therapeutics Policy Advisory Committee (Zimbabwe)

NNRTI Non-Nucleoside Reverse Transcriptase Inhibitor

NRTI Nucleoside Reverse Transcriptase Inhibitor

NVP Nevirapine

PI Principal Investigator

PCR Polymerase Chain Reaction

PMTCT Prevention of Mother to Child Transmission

PrEP Pre-exposure Prophylaxis

PSA Prostate-Specific Antigen

QA Quality Assurance

RCZ Research Council of Zimbabwe

RNA Ribonucleic Acid

RPR Rapid Plasma Reagin

SAE Serious Adverse Events

SMC Safety Monitoring Committee

SSC Scientific Steering Committee

STI Sexually Transmitted Infection

T&M Time and Motion

TB Tuberculosis

TDF Tenofovir

TPHA Treponema Pallidum Hemaglutination Assay

USCF University of California, San Francisco

US United States

UZ-UCSF University of Zimbabwe-University of California Collaborative Research Programme

VMMC Voluntary Medical Male Circumcision

WHO World Health Organization

WHO-CHOICE World Health Organization-Choosing Interventions that are Cost Effective

3TC Lamivudine

PROTOCOL SUMMARY

| **Title:** | SAFER: The impact, feasibility, acceptability, and cost-effectiveness of safer conception strategies for HIV-discordant couples in Zimbabwe. |
| --- | --- |
| **Summary:** | A mixed-methods project to (i) measure uptake, adherence to, and satisfaction with different safer conception strategies, (ii) characterize attitudes and preferences for safer conception strategies, and (iii) estimate the costs and potential cost-effectiveness of different strategies for safer conception for HIV-discordant couples in Zimbabwe. |
| **Primary Objectives:** | 1. To measure and characterize i) uptake and ii) adherence to, and iii) satisfaction with safer conception strategies, as well as iv) retention in care, v) sexual behaviors, vi) pregnancy rates, vii) HIV transmission to partner and child, and viii) obstetric and ix) neonatal outcomes among participants receiving safer conception.  2. To characterize i) provider and patient attitudes and ii) preferences for safer conception strategies and the iii) decision-making process around choosing and iv) adhering (patients only) to safer conception strategies.  3. To measure i) actual costs for providing safer conception, and through the use of mathematical models ii) estimate the potential cost-effectiveness of safer conception strategies in this setting. |
| **Population:** | Up to 80 HIV-discordant couples (50% with HIV-positive female partner) who are of reproductive age and have expressed the desire to conceive will participate in the non-randomized interventional pilot study.  In-depth interviews will be conducted among 20 discordant couples who participated in the pilot study, and 10 health care providers (5 who participated in the pilot study, and 5 who did not). |
| **Study Duration:** | 30 months |
| **Subject Participation Duration:** | 14-26 months |
| **Estimated Time to Complete Enrollment:** | 4 months |

INVESTIGATORS AND INSTITUTIONAL AFFILIATIONS

**Co-Principal Investigators**

Joelle Brown PhD, MPH

Associate Professor

Department of Obstetrics, Gynecology & Reproductive Sciences

Department of Epidemiology & Biostatics

University of California, San Francisco

Felix Mhlanga, MBChB, MMed

Obstetrician Gynecologist

Lecturer, University of Zimbabwe College of Health Sciences

Site Leader, UZ-UCSF Spilhaus Clinical Research Site

**Co-Investigators**

Bismark Mateveke, MBChB MMed

Honorary Lecturer, University of Zimbabwe, College of Health Sciences

Specialist Obstetrician and Gynecologist

Ministry of Health and Child Care

Harare Central Hospital

Zvavahera Mike Chirenje, MD FRCOG

Professor of Obstetrics, Gynecology & Reproductive Sciences

University of Zimbabwe

Executive Director, UZ-UCSF Collaborative Research Programme

Serah Gitome MBChB, MPH

Research Officer

Centre for Microbiology Research

Kenya Medical Research Institute

Craig R. Cohen, MD MPH

Professor of Obstetrics, Gynecology & Reproductive Sciences

University of California, San Francisco

Founding Director, Family AIDS Care and Education Services (FACES)

Elizabeth Bukusi, MBChB, MMed, PhD, MPH
Deputy Director of Research and Training

Kenya Medical Research Institute

Co-Director, Research, Care and Training Program (RCTP)

Lynae Darbes PhD

Associate Professor of Medicine

Center of AIDS Prevention Studies

University of California, San Francisco

Jim Kahn, MD PhD

Professor of Global Health Economics

University of California, San Francisco

**Partners**

Jim Rooney, MD

Medical Officer

PrEP Studies
Gilead

# Abstract/summary

Pregnancy and the desire for children are common among HIV-infected individuals, and HIV-discordant couples face a difficult choice between attempting pregnancy and risking HIV transmission to their partners.[^1-4^](#_ENREF_1) Evidence-based guidelines and recommendations for safer conception have recently been developed for settings in sub-Saharan Africa to address this issue.[^5^](#_ENREF_5) These guidelines have outlined various safer conception strategies for HIV discordant couples who desire conception, including initiation of antiretroviral therapy at any CD4 count, viral load monitoring and suppression, limiting condomless intercourse to the fertile period, semen washing, and artificial vaginal insemination. However, to date, no studies have been done in Zimbabwe or elsewhere to evaluate the feasibility of and preferences for these strategies among providers and patients. Moreover, no studies have been done to evaluate the potential cost-effectiveness of the different strategies for safer conception alone or in combination, on HIV prevention. This project will address this critical gap in HIV prevention. We will enroll and follow up to 80 HIV discordant couples who desire conception, and offer them strategies for safer conception.

Through this pilot study, we will measure and characterize i) uptake and ii) adherence to various safer conception options, as well as iii) retention in care, iv) sexual behaviors, v) pregnancy rates, vi) HIV, and vii) obstetric and viii) neonatal outcomes. We will also conduct in-depth interviews with providers and patients to characterize i) provider and patient attitudes and ii) preferences for safer conception strategies and iii) the decision-making process around choosing and iv) adhering (patients only) to safer conception strategies. Lastly, through costing studies conducted during the cohort pilot study, we will measure i) actual costs for providing safer conception, and through the use of mathematical models ii) estimate the potential impact and cost-effectiveness of safer conception strategies in this setting. Results from this study will potentially inform safer conception strategies in Zimbabwe, and may serve as a model for other countries that want to estimate the impact and cost-effectiveness of safer conception strategies in their settings.

# INTRODUCTION: BACKGROUND INFORMATION AND SCIENTIFIC RATIONALE

## Background Information

The reproductive needs of HIV-discordant couples who desire pregnancy represent an urgent public health problem that has been neglected in HIV prevention research. In Zimbabwe , and across sub-Saharan Africa, the vast majority of HIV-infected individuals are adults of reproductive age [^7^](#_ENREF_7). Pregnancy and the desire for children are common among HIV-infected individuals, and HIV-discordant couples face a difficult choice between attempting pregnancy and risking HIV transmission to their partners.[^1-4^](#_ENREF_1). Recent surveys have found that 30-50% of HIV-infected individuals in sub-Saharan Africa are involved in stable, HIV-discordant relationships,[^8-13^](#_ENREF_8), and HIV transmission within married, cohabitating HIV-discordant couples accounts for 44-60% of new HIV infections in some regions of sub-Saharan Africa.[^10^](#_ENREF_10)^,^ [^14^](#_ENREF_14)^,^ [^15^](#_ENREF_15). While knowledge of HIV discordance can lead to increased condom use, many discordant couples have unprotected intercourse often motivated by the desire to conceive.[^4^](#_ENREF_4)^,^ [^17^](#_ENREF_17)

In response to the urgent need for HIV prevention strategies to address the circumstances and reproductive goals of HIV-discordant couples, evidence-based recommendations for safer conception among HIV-discordant couples have recently been developed in several countries. These international guidelines [^5^](#_ENREF_5)^,^ [^18^](#_ENREF_18) reflect recent advances in antiretroviral therapy (ART), which has been shown to nearly eliminate HIV transmission to the uninfected partner[^19^](#_ENREF_19)^,^ [^20^](#_ENREF_20), and pre-exposure prophylaxis with emtricitabine/tenofovir disoproxil fumarate (PrEP), which has been shown to significantly reduce HIV transmission.

Currently, HIV discordant couples who attempt to conceive place themselves at considerable risk of transmission. This research study aims to help promote couples’ rights to conceive while at the same time decreasing the risk of HIV transmission. Therefore, even though safer conception strategies may not eliminate risk completely, they do offer a vast improvement over the status quo. In fact, some have argued that it would be unethical to withhold proven HIV prevention strategies to discordant couples who are trying to conceive.[^21^](#_ENREF_21)

## Safer conception strategies

There are several evidence-based strategies that can allow HIV serodiscordant couples to conceive while greatly minimizing HIV transmission risk to the uninfected partner (Table 1 below). These strategies are supported by cohort studies and randomized controlled trials.

**Table 1. Strategies available in resource-limited settings to reduce HIV transmission and allow couples to conceive**

| **Type of HIV-discordant couple** | **Safer Conception Strategy** | **Estimated HIV risk reduction** |
| --- | --- | --- |
| Either male or female is HIV infected  (goal: decrease sexual HIV transmission to the uninfected partner) | ART for infected partner  PrEP (oral, daily Truvada)  Treatment of STIs | 96%  63-75%  40% |
| Female HIV+/Male HIV-  (goal: decrease female to male HIV transmission) | Artificial vaginal insemination  Medical male circumcision | 100%  66% |
| Male HIV+/Female HIV-  (goal: decrease male to female HIV transmission) | Semen washing | 100% |

Early ART in the HIV-positive partner, and use of TDF-FTC pre-exposure prophylaxis (PrEP) in the HIV-negative partner reduces heterosexual HIV transmission in HIV-discordant couples, and these strategies have the potential to reduce HIV transmission while allowing conception to occur. Use of ART in the HIV-infected member of an HIV-discordant couple is associated with an 89-96% lower risk of HIV transmission to the uninfected partner. [^19^](#_ENREF_19)^,^ [^20^](#_ENREF_20) Use of TDF-FTC PrEP (‘Truvada’) in the HIV-uninfected partner has been shown to reduce HIV acquisition by 75%.[^22^](#_ENREF_22) Two studies from the developed world have provided evidence that use of ART and PrEP can prevent HIV transmission among HIV-discordant couples attempting conception. [^23^](#_ENREF_23)^,^ [^24^](#_ENREF_24) The majority of couples in these two studies conceived and there were no cases of HIV seroconversion in uninfected sexual partners. These results indicate that ART and PrEP have the potential to prevent HIV transmission in HIV-discordant couples desiring conception.[^25^](#_ENREF_25)

## Justification

Pregnancy is common among HIV-infected women in sub-Saharan Africa. An estimated 24% of births occurring in this region occur among HIV-positive women.[^26^](#_ENREF_26) According to the most recent Zimbabwe Demographic Health Survey, the overall fertility average is 3.6 children per woman, and antenatal HIV prevalence is 17.9%. Many have made the argument that reproduction is a basic human right, and that there is a need to ensure that HIV-affected individuals can safely conceive. [^21^](#_ENREF_21) International guidelines have shifted from recommending avoidance of pregnancy among HIV-affected populations to recognizing conception as a realistic option for HIV-affected couples.

However, no studies have yet been done in Zimbabwe to evaluate the impact, uptake, adherence to, and feasibility of delivering these strategies, or individual provider or patients’ preferences. Moreover, no studies have been done to evaluate the potential cost-effectiveness of the different strategies for safer conception alone or in combination on HIV prevention. This project will address this critical gap in HIV prevention. This study will enroll and follow up to 80 HIV discordant couples that desire conception, and offer them strategies for safer conception. Through this non-randomized interventional pilot study, we will measure and characterize i) uptake and ii) adherence to various safer conception options, as well as iii) retention in care, iv) sexual behaviors, v) pregnancy rates, vi) HIV, and vii) obstetric and viii) neonatal outcomes. We will also conduct in-depth interviews with providers and patients to characterize i) provider and patient attitudes and ii) preferences for safer conception strategies and the iii) decision-making process around choosing and iv) adhering (patients only) to safer conception strategies. Lastly, through costing studies conducted during the cohort pilot study, we will measure i) actual costs for providing safer conception, and through the use of mathematical models ii) estimate the potential impact and cost-effectiveness of safer conception strategies in this setting. Results from this study will inform provision of safer conception strategies in Zimbabwe, and may serve as a model for other countries that want to estimate the impact and cost-effectiveness of safer conception strategies in their settings.

# objectives

## Study Hypothesis

This study will explore the following hypotheses:

1. **Objective 1, Pilot Study:** We hypothesize that feasibility, measured by uptake of, adherence to, retention in care, and patient satisfaction with various safer conception strategies, will vary according to the type of safer conception strategy adopted, and will be influenced by participant characteristics (e.g. age, sex, pregnancy rates, relationship commitment, stigma, and beliefs about medication and reproductive-decision making)
2. **Objective 2, Qualitative Interviews**: We hypothesize that environmental and individual factors such as clinic characteristics, provider characteristics, and couple characteristics will influence acceptability, decision-making around, and perceived efficacy of safer conception strategies among patients and health care providers.
3. **Objective 3, Costing Study**: We hypothesize that provision of safer conception strategies for HIV-discordant couples desiring conception is a cost-effective method of preventing HIV transmission compared to the status quo (not providing safer conception strategies).

## General Objectives

The objectives of this study are to conduct a mixed methods (qualitative, quantitative, and cost-effectiveness) study to estimate the feasibility, acceptability, and potential cost-effectiveness of safer conception strategies in Zimbabwe

## Specific Objectives and Study Outcome Measures

**Objective 1, Pilot Study:** To measure and characterize i) uptake and ii) adherence to, and iii) satisfaction with safer conception strategies, as well as iv) retention in care, v) sexual behaviors, vi) pregnancy rates, vii) HIV, and viii) obstetric and ix) neonatal outcomes among couples receiving safer conception.

**Objective 2, Qualitative Interviews**: To characterize i) provider and patient attitudes and ii) preferences for safer conception strategies and the iii) decision-making process around choosing and iv) adhering (patients only) to safer conception strategies. (*Objective 2 will be informed by the clinical pilot study, and will be conducted after participants exit from the clinical pilot study).*

**Objective 3, Costing Study**: To measure i) actual costs for providing safer conception, and through the use of mathematical models ii) estimate the potential cost-effectiveness of safer conception strategies in this setting. *(Objective 3 will be informed by the clinical pilot study, and will be conducted after participants have begun enrolling into the clinical pilot study).*

## Sample size considerations

**Objective 1, Pilot Study:** The sample size for the pilot study (80 discordant couples) is based on feasibility rather than specific hypothesis testing. A sample of 80 discordant couples will allow us to generate reasonably precise 95% confidence intervals around major outcomes of interest (e.g. estimates of uptake of difference strategies, adherence, retention in care, pregnancy rates).

**Objective 2, Qualitative Interviews**: The sample size of the qualitative interviews (20 HIV-serodiscordant couples and 10 health care providers in Harare and Chitungwiza) has been chosen to allow us to reach saturation and redundancy in qualitative studies.[^31^](#_ENREF_31) The responses from 20 couples and 10 HCP will allow us to thoroughly examine the categories and themes that emerge from the discussions and also explore them in subsequent discussions with study participants.

**Objective 3, Costing Study**: The sample for the costing study will consist of the study staff and the 80 discordant couples that are participating in the pilot study.

# design and methodolOgy

## Study Site Background

Zimbabwe, situated in Southern Africa, is at the epicenter of the global HIV pandemic and continues to be extraordinarily burdened by HIV/AIDS, with a prevalence of 13.1%, and an incidence rate of approximately 3%. In Harare, the country’s largest city and its capital, HIV prevalence approximates the national rate of 13.1%, and incidence rate between 3%-3.5%, among adults aged 15-49 years old. In Zimbabwe, 11.4% of couples are HIV-discordant, of which approximately half are HIV-infected females married or cohabitating with an HIV-uninfected man. The overall fertility average is 3.6 per woman, and the antenatal HIV prevalence is 17.9%. While for some individuals HIV may reduce child bearing desires, over one third (37.2%) of HIV-positive women in Zimbabwe want more children. Strategies to prevent HIV transmission during attempts to conceive are needed in this setting.

UZ-UCSF, a collaboration between the University of Zimbabwe (UZ) and the University of California, San Francisco (UCSF) and led by Dr. Zvavahera Mike Chirenje, was founded in 1994 and has established seven clinical research sites in Harare and the surrounding communities. More than 5,000 men, women, and children have been enrolled in studies for the prevention and treatment of HIV/AIDS.

All clinic activities will take place at UZ-UCSF’s Zengeza Clinical Research Site, which is located within Zengeza Municipality Clinic in Chitungwiza.  The municipality clinic serves as the first medical point of entry for adults and children for treatment of acute and chronic illnesses, maternity care, family planning, and well-baby services. 41% of the local population is comprised of adults over 15 years of age, among whom 24% are women of child-bearing age (15-49 years).  HIV prevalence among antenatal clinic attendees is 16%; the prevalence of HIV-infection among women is 18%, and is 12% among men.

## Study Design

There will be three components to this study. First, we will conduct a non-randomized interventional pilot study. The pilot study will be followed by cross-sectional qualitative interviews among a sample of providers and patients participating in the pilot study. Third, we will conduct costing studies during the pilot cohort study to estimate the costs associated with delivering the study.

During the non-randomized interventional pilot study, HIV-discordant couples will be counseled on and offered strategies for safer conception. Specifically, participating couples will:

1. Receive HIV counseling and testing
2. Be screened and treated for genital tract infections, and women will be screened for cervical cancer
3. Be screened for anemia (women)
4. Receive intensive counseling to prevent HIV transmission to partner and baby
5. Receive counseling on identifying the time of ovulation
6. Choose and initiate *at least one* of the following effective HIV prevention strategies:
   - ART therapy (and CD4 level) and viral load monitoring in the HIV-infected partner. This strategy is coupled with condomless sex timed to the fertile period after viral suppression;
   - PrEP in the HIV-negative partner. This strategy is coupled with condomless sex timed to the fertile period;
   - Semen collection with artificial vaginal insemination at the time of ovulation (among couples with an HIV-positive woman/HIV-negative man).
   - Semen washing with intrauterine insemination (IUI) at the time of ovulation (among couples with HIV-negative woman/HIV-positive man)
7. Counseled to use male condoms 100% of the time, except for couples choosing ART or PrEP who will have condomless sex only during peak fertile days. Male condoms will be provided free of charge throughout the study
8. Receive regular pregnancy testing and HIV testing
9. Be offered nutritional counseling and folic acid supplementation (women)

Though we have considered a randomized design for this pilot study, ethical considerations preclude denying HIV prevention interventions to some. Moreover, allowing couples to make informed choices and to select their preferred strategies may lead to increased adherence [^33^](#_ENREF_33). In this pilot study, all couples will be counseled on the range of safer conception strategies, provided with expert consultation so that strategies can be tailored to their specific needs, which may vary from couple to couple (see Section 5.6), and will be allowed to choose more than one strategy if they want (for example, ART with viral load monitoring in the HIV-positive partner plus artificial vaginal insemination).

## Study Participants

**Objective 1, Pilot Study:** We will enroll up to 40 HIV-discordant couples who desire conception into the pilot study. Couples are defined as sexual partners of the opposite sex who are married, living together, or otherwise consider each other a primary partner and plan to be together for at least one year. There will be an equal number of men and women in the study (up to 80 men and 80 women), and we will ensure that approximately half of HIV-positive participants are female. We believe this study will be more useful in guiding policy and future scale up of safer conception if both female positive/male negative and female negative/male positive couples are included. All participants will be over 18 years of age and women will be 18-35 years of age to maximize the possibility of successful conception. All participants will be in good health and HIV positive participants will not have any AIDS-defining illnesses.

During the pilot study, participants will be provided with clinical consultation and care as required by participants, clinical and laboratory testing and treatment, as needed, HIV care and treatment for index participants during and upon completion of study participation; and antenatal, obstetric, and neonatal care. Participants will be offered transport reimbursement for their participation, equivalent to approximately U.S. $10 per visit per participant.

**Objective 2, Qualitative Interviews:** 20 HIV-discordant couples who participated in the pilot study, and 10 health care providers (5 who provided safer conception services in the pilot study and 5 who did not) will be purposively selected for participation in qualitative interviews. Members of couples will be interviewed separately. Participants will be offered a transport reimbursement for their participation, equivalent to approximately U.S. $10 per participant.

**Objective 3, Costing studies:** For the costing studies, study staff will observe clinical activities to track how much time clinical staff actually spends delivering the interventions, in order to calculate costs. However, data collection methods will impose no risk or burden on human subjects.

## Description of Safer Conception Strategies to be offered

All discordant couples will be counseled about the risks of HIV transmission and available options for reducing the risk of HIV transmission while allowing conception to occur, and offered safer conception strategies. Participants will be counseled that no single method (including treatment of the infected partner) is fully protective against transmission of HIV, and cautioned about the potential risk of transmission of HIV to the uninfected partner and to their offspring [^34^](#_ENREF_34).

All couples will:

1. Be screened for anemia (women)
2. Be screened and treated for genital tract infections, and women will be screened for cervical cancer
3. Receive intensive counseling to prevent HIV transmission to partner and baby
4. Receive counseling on identifying the time of ovulation
5. Receive counseling to use male condoms 100% of the time, except for couples choosing ART or PrEP who will have condomless sex only during peak fertile days. Male condoms will be provided free of charge throughout the study
6. Be offered nutritional counseling and women will be offered folic acid supplementation
7. Receive monthly HIV counseling and testing until pregnancy, and quarterly thereafter with appropriate management if individual becomes infected
8. Receive monthly pregnancy testing until pregnancy occurs, and quarterly thereafter

In addition, all couples will be counseled on the range of HIV prevention strategies, provided with expert consultation by a study clinician so that strategies can be tailored to the couples’ specific needs, which may vary from couple to couple and will depend on whether the HIV-positive partner is male or female.

Specific HIV prevention strategies are described below:

**Male HIV Infected/Female HIV-negative:**

*For the HIV-positive male partner:*

Pre-conception:

- ART with intensive adherence support and viral load monitoring with the goal of viral suppression. Adaptation of regimen as needed. This strategy is coupled with counseling on condomless sex timed to the fertile period.
- Counseling on semen collection at home or clinic for semen washing and IUI at the time of ovulation

During pregnancy:

- Counseling on 100% condom use
- Continued ART with intensive adherence support

*For the HIV-negative female partner:*

Pre-conception:

- Daily oral PrEP with intensive adherence support. This strategy is coupled with counseling on condomless sex timed to the fertile period.
- Counseling on semen collection at home or clinic for semen washing and IUI at the time of ovulation

During pregnancy:

- Counseling on 100% condom use
- Counseling and consenting for continued PrEP use in pregnancy
- HIV counseling and testing during pregnancy with appropriate management if female partner becomes infected, and prevention of mother-to-child transmission.

**Female HIV Positive/Male HIV-negative:**

*For the HIV-positive female partner:*

Pre-conception:

- ART with intensive adherence support and viral load monitoring with the goal of viral suppression, and adaptation of regimen as needed. This strategy is coupled with counseling on condomless sex timed to the fertile period.
- Counseling on semen collection for artificial vaginal insemination at home at the time of ovulation

During pregnancy:

- Counseled on 100% condom use
- Counseled on and provided with ART during pregnancy for prevention of mother-to-child transmission

*For the HIV-negative male partner:*

Pre-conception:

- Counseling on semen collection for artificial vaginal insemination at home at the time of ovulation
- Daily oral PrEP with intensive adherence support. This strategy is coupled with condomless sex timed to the fertile period.

During pregnancy:

- 100% condom use
- HIV counseling and testing during pregnancy with appropriate management if male partner becomes infected
- Counseled on and offered continued use of PrEP

**ART with viral load monitoring**

Use of ART in the HIV-infected member of an HIV-discordant couple is associated with an 89-96% lower risk of HIV transmission to the uninfected partner.[^19^](#_ENREF_19)^,^ [^20^](#_ENREF_20) The HPTN 052 study[^20^](#_ENREF_20) found that early initiation of ART, in which the HIV-infected partner has ≥350 CD4 cells/μL, resulted in a 96% reduction in HIV transmission among discordant couples. A study in Spain of HIV-serodiscordant couples desiring conception found that use of ART and viral load monitoring allowed conception and prevented HIV transmission; among 62 HIV-discordant couples with the HIV-positive person on ART, 76 natural pregnancies occurred over a 12 month period, 68 children were born, and there were no cases of HIV seroconversion in uninfected sexual partners.[^23^](#_ENREF_23)

In this study, HIV positive participants will be counseled on use of ART for the prevention of HIV transmission, the importance of adherence, and potential side effects. ART regimens will be consistent with current Zimbabwe national treatment guidelines. After participants are found to have suppressed viral load, they will be counseled on attempting conception. Couples with suppressed viral load will be instructed to have condomless intercourse 2 days prior to ovulation, on the day of ovulation and 2 days after ovulation for up to 12 months or until conception occurs.

Participants taking ART will receive adherence counseling based on established adherence protocols in use at our study site. These involve establishing patient rapport, involving patients in their care planning, HIV and ART education, assessing individual barriers to adherence, and checking for support systems in place. Counselors then work with patients to establish a plan to promote adherence, including establishing treatment buddies and family caregivers, preferred times of day to take medicines and identifying reminder systems. Participants who are found to have <95% adherence during follow-up will receive additional adherence counseling.

ART and viral load monitoring will be provided for up to 12 months or until conception. After conception, men and women who are taking ART will be counseled on the risks and benefits of continuing ART during pregnancy, and encouraged to continue ART during pregnancy. All couples will be counseled to continue with 100% condom use during and after pregnancy. HIV care and treatment for HIV positive participants will continue after completion of study participation and will be provided through MOH clinics.

**PrEP**

The Partners PrEP trial, conducted among 4,758 HIV-discordant couples in Kenya and Uganda demonstrated that daily TDF-FTC (Truvada) reduced the overall risk of HIV acquisition by 75%.[^35^](#_ENREF_35) PrEP was found to be safe and well tolerated by participants in this trial. A study in Italy of HIV-discordant couples attempting conception found that PrEP in the HIV negative partner, in combination with ART in the HIV positive partner, can prevent HIV transmission[^24^](#_ENREF_24); among 46 HIV discordant couples using PrEP, the majority (75%) conceived within 6 months, and there were no cases of HIV seroconversion in uninfected sexual partners, and no adverse events.

In this study, HIV negative participants will be counseled on the use of daily PrEP (TDF-FTC ‘Truvada’), the importance of adherence, and potential side effects. PrEP will be prescribed for once-daily use.

Participants taking PrEP will receive adherence counseling based on established adherence protocols, as described above. Patient readiness is then assessed prior to PrEP initiation. Participants who are found to have <95% adherence during follow-up will receive additional adherence counseling.

Couples using PrEP as a safer conception strategy will be instructed to have condomless intercourse 2 days prior to ovulation, on the day of ovulation and 2 days after ovulation for up to 12 months or until conception occurs. Daily oral PrEP will be provided for up to 12 months or until conception. After conception, HIV-negative men and women who are taking PrEP will be counseled on the risks and benefits of continuing PrEP during pregnancy, and allowed to choose whether to continue PrEP during pregnancy. Women who become pregnant while on PrEP will need to provide consent for PrEP continuation during pregnancy. All couples will be counseled to continue with 100% condom use during and after pregnancy. For this study, PrEP will be discontinued in HIV-negative women prior to breastfeeding. HIV-1 uninfected women will be encouraged to breastfeed their infants according to WHO and Zimbabwean national guidelines.

**Semen collection with artificial vaginal insemination**

Semen collection with artificial vaginal insemination at the time of ovulation consists of a man ejaculating into a condom and the contents are introduced into the vagina timed to peak fertility. This can be completed at home and confers zero HIV risk to the male partner.[^36^](#_ENREF_36) This option will be available to couples with HIV-negative male/HIV-positive female partner. For couples who choose this option, on the insemination days, semen will be collected using a non-lubricated condom and then the semen will be drawn into a sterile needle-less syringe at home. Women will then perform vaginal insemination with this collected semen. [^36^](#_ENREF_36) Couples will be instructed to perform vaginal insemination 2 days prior to ovulation, on the day of ovulation and 2 days after ovulation for up to 12 months or until conception occurs.

**Identification of the ovulation period**

Couples will be instructed to identify ovulation, the menstrual cycle midpoint, by maintaining a calendar and assessing the consistency of cervical mucus. [^37-39^](#_ENREF_37) A calendar will be provided to each couple to assist them with predicting ovulation.

**Semen washing**

Couples with an HIV positive male/HIV-negative female will be counseled on semen washing as an option. Semen washing ^58^ is the process in which individual sperms, which do not contain HIV, are separated from the seminal fluid, which does contain HIV. The remaining sperm is suspended in a substitute medium and inserted into the woman’s uterus using the intrauterine insemination (IUI) technique at the time of ovulation. This method confers minimal HIV risk to the female partner; to date, there have been no reports of seroconversion following sperm washing with IUI. ^59^ For couples who choose this option, on the day of ovulation, or up to one week prior to ovulation, semen will be collected using either masturbation at the clinic or at home or through sexual intercourse at home. Semen will be delivered to the laboratory within one hour of collection for semen washing. Sperm samples collected on the day of ovulation will be inserted into the woman’s uterus using IUI at the study clinic. Sperm samples collected prior to ovulation will be frozen with liquid nitrogen according to the European Society of Reproduction and Embryology guidelines (ESHRE) ^60^ and thawed sperm will be inserted into the woman’s uterus using IUI on the day of ovulation at the study clinic.

# eligibility, ENROLLMENT AND PROCEDURES for NON-RANDOMIZED INTERVNETIONAL Pilot study (Objective 1)

## Inclusion and Exclusion Criteria

***Inclusion criteria***

**For all couples:**

- Couple expresses a desire to conceive
- Sexually active (defined as having vaginal sex with one another at least 6 times in the past 3 months
- Willing to enter the study as a couple and intending to remain as a couple and have a sexual relationship for the next 12 months
- Willing to use at least one of the following strategies for safer conception (either ART with viral load monitoring in HIV positive partner, PrEP in HIV negative partner, artificial vaginal insemination among couples with an HIV positive female/HIV negative male, or semen washing with intrauterine insemination among couples with an HIV-positive male/HIV-negative female)
- HIV-discordant couples who participated in the pilot study (for qualitative interviews)
- Willing to participate in one-on one discussions regarding safer conception strategies (for qualitative interviews)
- Able and willing to provide adequate locator information for study retention purposes, as defined by study standard operating procedures;
- For men, age ≥18 years. For women, age 18 - 35 years;
- Able and willing to provide written informed consent (illiterate participants will append a thumbprint to the consent document after being consented in the presence of an independent witness)
- No active and serious infections, including active tuberculosis infection; active clinically significant medical problems including cardiac disease (e.g., symptoms of ischemia, congestive heart failure or arrhythmia), pulmonary disease (steroid-dependent chronic obstructive pulmonary disease), and previously diagnosed malignancy expected to require further treatment.

**For HIV-uninfected members of the couple**

- HIV-negative based on parallel negative HIV rapid tests, both at study screening and enrollment visit
- Adequate renal function, defined by normal creatinine levels and estimated creatinine clearance ≥60 mL/min
- Not currently breastfeeding a child under 18 months of age
- Not currently using PrEP
- Not amenorrheic

**For HIV-infected members of the couple**

- HIV-positive based on parallel positive HIV rapid tests, based on national algorithm
- Not currently pregnant
- No current AIDS-defining illness
- *- Note: current breastfeeding is permitted for HIV-infected partners*

***Exclusion criteria***

**For couples**

- History of infertility defined as a year or more of regular unprotected intercourse with current partner without pregnancy, or otherwise medically diagnosed infertility
- Unable or unwilling to provide written informed consent
- Currently on any concomitant medication that requires the participant to avoid use of PrEP

## Strategies for Recruitment and Retention

**Community preparedness.** In order to build community awareness and foster preparedness for the study, we will establish a Community Advisory Group consisting of local **stakeholders from the community, such as people living with HIV, youth, local health providers, and local leaders. We will also work with the** UZ-UCSF Community Department**, community mobilizers, and public health officers who engage and educate community members on HIV prevention, safer conception strategies, and other health-related information related to the program.**

**Identification and recruitment of participants.** The study site will be located in Chitungwiza at Zengeza Clinical Research Site. Our programme has enrolled over 1,000 HIV-discordant couples. We will use approaches that have been successfully employed in recruiting discordant couples within our research programme. This includes community preparedness and mobilization, working with HIV Counseling and testing (HCT) centers to refer newly diagnosed discordant couples to Zengeza Clinical Research Site, training and support of “couples ambassadors” to encourage couples to undergo HCT, and linking HIV testing services with HIV care and treatment programs in the community, a community advocacy and mobilization model that has been successful elsewhere [^4^](#_ENREF_4)^,^ [^41^](#_ENREF_41).

**Retention of study participants.** In order to ensure high retention of study participants, we will employ the following techniques that have proven successful in our research among discordant couples in this region. Late participants will be contacted by study staff immediately. Participants will be encouraged to come to the study clinic at any time for advice, or for further information. Logistical arrangements will facilitate couple attendance at clinic visits and counseling sessions. Services will be offered six days a week in order to align with findings that men desire flexible study schedule.

## Screening visit

Screening visits will determine eligibility for HIV-discordant couples referred to the study. Study staff will provide information about the study, including screening visit procedures, risk and benefits, and each partner in each couple will provide independent written informed consent for screening.

Following informed consent, the study nurse will administer a screening questionnaire designed to assess the participant’s socio-demographic history, medical history and use of HIV medications, and a targeted physical exam will be performed. Obstetric history will be collected, and syndromic management for sexually transmitted diseases will be conducted, and women will be evaluated for cervical cancer.  If, after questionnaire administration and physical exam, none of the exclusion criteria have been met, a blood specimen will be collected and sent immediately for rapid HIV tests to confirm HIV status, as well as measure CD4 levels in the HIV-positive participant. Participants will be screened for anemia. Urine will be collected to test for proteinuria in HIV+ positive individuals. Treatment for sexually transmitted diseases will be provided per syndromic management guidelines.

Participants will be scheduled to return for their results and determination of study eligibility between 2-4 weeks after the screening visit, and to coincide with Days 5-12 of menses.

Uncircumcised HIV-negative men will also be counselled about the benefits of voluntary medical male circumcision (VMMC) and referred to Health Centres where VMMC is available free of charge. Men will need to wait at least 6 weeks after the circumcision procedure before having intercourse. After healing or 6 weeks post-circumcision (whichever comes later), these men and their female partners will be rescreened to confirm study eligibility.

## Enrollment visit and 2-month run-in period

**At the enrollment visit,** informed consent for enrollment in the study will proceed when eligibility is confirmed. HIV negative participants will have repeat HIV antibody counseling and testing to confirm seronegative status, and women will be rescreened for pregnancy to determine eligibility. Women with evidence of precancerous lesions or cancer of the cervix will be referred for further management. After study procedures are explained, each partner will be asked to complete a comprehension questionnaire designed to evaluate understanding of study participation. If the couple demonstrates comprehension of the study procedures and potential risks, each member of the couple will be asked to provide independent written informed consent for participation in this study.

**The enrollment visit (M0)** marks the beginning of the 2-month run in period. At this visit, both members of the couple will be interviewed and a structured questionnaire will be completed to assess beliefs about safer conception strategies, including likelihood of HIV transmission [^42^](#_ENREF_42), reproductive decision-making [^43^](#_ENREF_43), relationship commitment [^44^](#_ENREF_44) and power [^45^](#_ENREF_45), sexual behaviors, including condom use and concurrent partnerships, and HIV-associated stigma [^46^](#_ENREF_46).

Enrolled couples will be followed monthly for 2 months before initiating conception attempts. The purpose of this 2-month run-in period is to provide couples with intensive couples-based counseling about HIV risk reduction in the context of trying to conceive. In addition, couples will be taught to follow the woman’s menstrual cycles to determine the fertile period of her cycle. Participants will be counseled to use condoms 100% of the time during the run-in period, and condoms will be provided free of charge.

At the Enrollment visit (Month 0), clinical data will be collected, and will include prior medication toxicities and other adverse events (AE), current medications and herbs taken, and medical history.

Female partners will collect a vaginal swab to detect the presence of prostate specific antigen (PSA), a validated measure of consistent condom use.

Blood will be collected to test for primary and latent syphilis infection. HIV positive participants will have an HIV viral load test. A blood sample from HIV-1 uninfected partners will be collected to be used for retrospective PCR testing if the participant seroconverts early during follow up.

At this visit, participants will be provided with intensive counseling to enhance HIV prevention to partner and baby. All participants will be offered nutritional counseling and women will be offered folic acid supplementation.

All participants will receive counseling on determining their fertile days, and a fertile days calendar will be provided. Couples will be taught to follow the woman’s menstrual cycles by maintaining a calendar and evaluating the consistency of her cervical mucus to determine the fertile period of her cycle.

Couples will also be counseled on their options for safer conception. Couples who opt for ART and viral load monitoring will be provided with ART (according to NMTPAC/MOHCC guidelines) irrespective of CD4 count, and asked to abstain from condomless sexual intercourse until the HIV RNA in the HIV-infected partner is found to be <50 copies/mL and after two months of charting their menstrual cycle/fertile days. Participants taking ART will have viral load tested at the enrollment visit and monthly after initiation of ART until conception, and then quarterly thereafter. When the HIV-infected participant’s HIV RNA is <50 copies/mL, the couple will be notified that they can attempt conception. It is anticipated that most participants will have an undetectable viral load HIV RNA after two months of consistent ART use, and that most couples will be able to initiate safer conception attempts at Month 3 (M3).

Couples who opt for PrEP will be provided with once daily PrEP (Truvada), and asked to abstain from condomless sexual intercourse until after the M3 visit, after two months of taking PrEP and two months of charting their menstrual cycle/fertile days. It is anticipated that most participants will consistently use PrEP, and be able to initiate safer conception attempts at Month 3 (M3). Participants who report less than 95% adherence to PrEP over the past month at M3 visit will be asked to delay conception attempts until consistent PrEP use is achieved.

Couples who opt for artificial vaginal insemination or semen washing with intrauterine insemination will be asked to abstain from condomless sexual intercourse until M3, after two months of charting their menstrual cycle/fertile days.

Consistent adherence to safer conception strategies will be important to prevent HIV transmission, and for HIV-infected female participants, to reduce the risk of MTCT of HIV. Participants will receive adherence counseling based on the established adherence protocols at our site, which involve establishing patient rapport, involving patients in their care planning, strategy education, assessing individual barriers to adherence, and checking for support systems in place. Counselors then work with patients to establish a plan to promote adherence, including establishing treatment buddies and family caregivers, preferred times of day to take medicines and identifying reminder systems. Participants who are found to have <95% adherence during follow-up will be referred immediately for additional adherence counseling.

All participants will be instructed to not attempt conception until they are notified by study staff, which we anticipate will be at the end of the two month run in period.

Participants will be scheduled to return to the clinic in one month (M1) to coincide with Days 5-12 of menses.

**At the Month 1 (M1) and Month 2 (M2) visits,** HIV negative participants will have a blood draw for repeat HIV antibody counseling and testing to confirm seronegative status. HIV positive participants will have a repeat viral load test (at M2 visit only). Fertile days calendars will be reviewed, and additional counseling on determination of fertile days will be conducted, as needed. A small hair sample will be collected from all participants who have hair and are taking ART or PrEP. Participants who do not have hair to cut from their head at their scheduled study visit, will have hair collected from them as soon as they have hair. Women will collect a vaginal swab to detect the presence of prostate specific antigen (PSA), a validated measure of consistent condom use, and be tested for pregnancy. At the end of the M1 and M2 visit, participants will be scheduled to return to the clinic to coincide with Days 5-12 of menses of their next menstrual cycle.

Participants will be assessed for adherence to ART, PrEP and condom use. For participants taking ART or PrEP, adherence will be estimated through i) three day self-report, ii) viral load (for ART only), and iii) detection of antiretroviral medication (ART and PrEP) in a small hair sample. At each follow-up visit study staff will assess participant adherence through a structured questionnaire that queries the number of missed doses over the preceding three days. Excellent adherence will be defined as ≥95% of doses taken. This threshold was chosen because it correlates well with viral suppression [^47^](#_ENREF_47)^,^ [^48^](#_ENREF_48) and is the threshold most frequently used in adherence studies in sub-Saharan Africa [^49^](#_ENREF_49). For participants who choose artificial vaginal insemination or semen washing, adherence will be estimated through self-reported condom use and PSA test results. Participants will be evaluated for possible causes of poor adherence including medication side effects, difficulty with dosing schedule, difficulty with following technique, and stigma [^50^](#_ENREF_50)^,^ [^51^](#_ENREF_51).

## Monthly Follow-up Visits before conception occurs

Following the 2-month run in phase, study visits will occur monthly for all participants until conception occurs, then quarterly thereafter (Please see APPENDIX A: Schedule of Events Schedule of Screening, Enrollment/Run-In, and Delivery of Strategies**)**. At each visit, couples will be evaluated for adherence to their safer conception strategies. Participants taking antiretrovirals will be evaluated for tolerance.

At each visit, female partners will collect a vaginal swab to detect the presence of prostate specific antigen (PSA), a validated measure of consistent condom use, and be tested for pregnancy. Participants taking antiretrovirals (ART or PrEP) will provide a hair sample for batched adherence testing.

HIV positive participants will be evaluated for viral load. HIV-negative participants will have HIV antibody counseling and testing.

Couples will be counseled to use condoms 100% of the time, except for couples choosing ART or PrEP who will have condomless sex timed to peak fertile days. On-going couples-counseling sessions on HIV risk reduction will occur quarterly.

After conception occurs, couples will be counseled and encouraged to reinitiate 100% condom use to further reduce the risk of HIV transmission. Condoms will be provided to the participants at no cost.

**Figure 1. Schema and follow-up time for Pilot Cohort Study (Objective 1)**

**Adverse events.** For the purposes of this study, only serious adverse events (SAEs), and adverse events felt related to PrEP, ART, vaginal insemination, and semen washing will be documented on case report forms. Serum creatinine will be monitored at Month 1 and then every 6 months; serum creatinine elevation of Grade 1 or greater that is confirmed on a second sample drawn for follow-up of an abnormal result will result in temporary hold of PrEP. A participant with a Grade 2 or higher creatinine or a calculated creatinine clearance of <50 mL/min will have PrEP temporarily withheld, and a second sample will be drawn for confirmation; confirmed Grade 2 or higher creatinine events or confirmed creatinine clearance <50 mL/min will result in permanent PrEP discontinuation.

SAEs felt to be related to PrEP, ART, vaginal insemination, or semen washing will result in temporary hold of that method of safer conception. In the case of temporary holds, the hold will continue until the event is stabilized or resolved. If the event resolves, PrEP, ART, vaginal insemination, and semen washing may be reinitiated at the discretion of the Investigator, resuming safety monitoring. We will use the Division of AIDS Toxicity Table for Grading Severity of Adult and Pediatric Adverse Events (DAIDS AE Grading Table) to evaluate AEs in participants and infants born to participants. If Grade 3 or 4 toxicity is documented following the dosing of any antiretrovirals, study staff will assess for alternate explanations for clinical and laboratory abnormalities. For laboratory abnormalities, repeat laboratory testing will be performed within 72 hours. Study staff will follow participants with abnormal clinical findings and laboratory values at least weekly until the toxicity resolves to less than Grade 2 or no further improvement is anticipated.

Study staff will receive training in the identification and management of AEs. In addition, we will educate participants about potential AEs associated with prescribed ART and PrEP regimens and will encourage them to present to the study clinic for all potential AEs affecting them or their infants. The study clinic will maintain a 24-hour, 7 days per week telephone line so that participants may report with potential AEs as they arise.

**Detectable viral load during follow-up.** Participants who are taking ART who have detectable HIV RNA (> 50 copies/mL) following viral suppression will be referred to an ART counselor immediately for adherence counseling. They will also be advised to resume 100% condom use. When the HIV-infected participant’s HIV RNA is <50 copies/mL, the couple will be notified that they can resume conception attempts. Participants with detectable HIV RNA will be medically managed following the Zimbabwean ART guidelines.

**Discontinuation of safer conception strategies.** Safer conception strategies will be discontinued under any of the following circumstances: 1) the study participant voluntarily chooses to discontinue; 2) the administration of the strategies is terminated by the PI, as may occur if the participant is judged by the investigator to be at significant risk of failing to comply with the provisions of the protocol as to cause harm to self or seriously interfere with the validity of the study results; 3) participants who do not conceive after 12 months will be discontinued from strategies and offered an infertility evaluation. In each case, a discontinuation form will be completed. This evaluation will include a structured interview in which clinical symptoms, including AEs and risk factors will be assessed. Reasons for discontinuing strategies will be assessed and recorded. Non-adherence to study strategies will not lead to discontinuation of study participation (see above). Participants who no longer wish to continue following safer conception strategies and their partners will be encouraged to continue study follow-up through the end of the 12 month follow-up period, including risk reduction counseling, 100% condom use, and quarterly HIV testing for the seronegative partner.

## Quarterly Follow-up Visits after conception occurs

After conception occurs, couples will visit the clinic quarterly (Please see Appendix A. Schedule of Events). HIV-negative women who were using PrEP will be counseled about the risks and benefits of continuing PrEP through pregnancy and will provide informed consent if they choose to continue PrEP. At each quarterly visit, staff will perform a targeted physical exam, assess adherence to ART and PrEP, and obtain blood for HIV viral load for HIV positive participants, and an HIV antibody test for HIV-negative participants. HIV positive participants taking ART will have CD4 testing once every 6 months, per Zimbabwean guidelines.

**Routine antenatal care**. Women who conceive will be referred to the ANC clinic of their choice in order to receive routine antenatal care in accordance with the Focused Antenatal Care (FANC) package developed by the WHO and adopted by the Zimbabwean Ministry of Health [^52^](#_ENREF_52). We review and abstract data from their ANC card at each study visit. If women are not being provided with standard FANC package, the study staff will provide supplemental clinical care as needed (e.g. folic acid supplementation). All pregnant participants will be advised to deliver in a health facility.

**Follow-up visits after delivery.** Following delivery, routine maternal and child visits will occur at the Maternal and Child Health Clinic according to the national guidelines. Study visits for participants and children will occur 6 weeks and 12 weeks following delivery (Please see Appendix B. Schedule of Events in Pregnancy and Postpartum). Data will be abstracted from the medical records of the participants and their children. Infants born to mothers with HIV will be monitored for intolerance of ART prophylaxis and blood will be collected from infants for HIV DNA testing at six weeks and 12 weeks postpartum. HIV-exposed infants will be treated according to the Infant ARV prophylaxis as per Zimbabwe guidelines.

**Participants who do not conceive**. Couples who do not conceive after 12 months of attempted conception will be referred to a health facility with gynecological care for fertility evaluations, including hystersalpinography and semen analysis.

**Participants who have a miscarriage.** Couples who conceive but have a miscarriage will be allowed to rescreen and re-enroll.

**HIV seroconversion**. Although we anticipate few HIV seroconversions during the study, HIV-negative participants who have a reactive HIV rapid antibody test during the study will be counseled that additional HIV testing is required, and blood will be drawn for confirmatory HIV serology and for HIV RNA level. Participants will be asked to return after 2 weeks to receive the results of confirmatory testing, and counseling, and if confirmed HIV positive, a blood sample will be collected from the recently infected partner for HIV genotypic drug resistance testing. The HIV-infected participant of a seroconverting partner participant will also provide a blood sample within 2 weeks of partner seroconversion for the purposes of phylogenetic analysis to determine if the transmission is linked to the HIV-positive partner.

## Interim Visits

Interim visits may occur at any time during the study for a variety of reasons. All interim contacts and visits will be documented in participants’ study record. When interim contacts or visits occur in response to participant reports of AEs, study staff will assess the reported event clinically, its relatedness to the study, and provide appropriate medical care or referrals.

## Visit Windows

For each monthly visit, the study window starts 15 days before their scheduled visit and ends 15 days after their scheduled visit. For each quarterly visit after conception, the study window starts 45 days before their scheduled visit and ends 45 days after their scheduled visit. For each study visit after delivery, the study window starts 21 days before their scheduled visit and ends 21 days after their scheduled visit.

## Study Withdrawal

It will be made clear to participants that they are free to withdraw from the study at any point without repercussions and will be given appropriate referrals for care. Non-adherence to safer conception strategies will not lead to discontinuation of study participation (see section 5.5 Discontinuation of Safer Conception Strategies).

## Laboratory Procedures/Evaluations

All specimen transport, processing, testing, and results reporting will be conducted in accordance with Good Clinical and Good Laboratory Practices (GCP and GLP). Our clinical laboratories in Harare and Chitungwiza have established standard operating procedures, including internal and external QA procedures for all protocol-specified laboratory tests. The following specimens will be tested at the local laboratory:

1. Blood for HIV viral load and CD4 T-cell counts, HIV antibody, syphilis serology, and infant dried blood spot DNA PCR
2. Urine for pregnancy testing, and urinalysis
3. Genital specimens for STI testing, and cervical cancer screening, if indicated
4. PSA testing of vaginal swabs

In the event that local capacity is not available for specialized testing, reasonable efforts will be made to build local capacity. In the event that local capacity is not warranted, samples will be exported with all required permissions and permits to external testing laboratories. The following specimens will be tested at UCSF due to unavailability of the testing facilities in Zimbabwe.

| 1. HIV-1 genetic sequencing (Plasma) | ARI-UCSF Laboratory of Clinical Virology, UCSF, Dr. Teri Liegler, Director | Determination of drug resistant mutations and viral sequence for phylogenetic measurements. |
| --- | --- | --- |
| 2. ART/PrEP adherence assessment (hair) | UCSF Hair Drug Studies Unit,  Dr. Monica Gandhi | Determination of drug concentration levels for an objective measure of |

Specimens requiring shipment to UCSF will be shipped in accordance with IATA regulations. Samples shipped to UCSF laboratories for testing will be destroyed immediately after the testing.

# PREP MEDICATION

This study will provide open-label PrEP (‘Truvada’) to HIV-1 uninfected individuals in HIV-1 discordant couples at no extra cost. Co-formulated FTC/TDF at 200 mg / 300 mg respectively will be used. The dose of FTC/TDF is the standard dose approved by the U.S. Food and Drug Administration.

It has been demonstrated that FTC/TDF tablets are stable at 25°C and 60% relative humidity for

up to 48 months, at 30°C and 65% relative humidity for up to 24 months, and at 40°C and 75%

relative humidity for up to 6 months. Additionally, tablets have been shown to be stable at 50°C

for up to 1 month.

FTC/ Tablets will be packaged as a one-month supply (30 tablets) in bottles with child-resistant screw caps. In addition to the tablets, each bottle contains a silica gel desiccant to protect the product from humidity and the polyester packing material that cushions it during handling and shipping. PrEP will be dispensed in an amount to last until the next scheduled visit – thus, at enrollment, a one-month supply (to last until the next scheduled monthly visit) until conception occurs, and a three-month supply thereafter (corresponding to quarterly visits).

The study drug will be stored in accordance with the drug manufacturer’s recommendations.

The pharmacy and storage facility will be controlled by the UZ-UCSF programme, and will have locked, climate-controlled environments, with controlled humidity and temperature to remain within limits allowed by the manufacturer for drug storage.

Concomitant medication use will be recorded. PrEP will be avoided, per investigator discretion,

in individuals receiving ongoing therapy with anti-retroviral medications, interferon (alpha, beta,

or gamma) or interleukin (e.g., IL-2) therapy, metformin, systemic aminoglycoside antibiotics,

amphotericin B, cidofovir, systemic chemotherapeutic agents, other agents with significant

nephrotoxic potential, other agents that may inhibit or compete for elimination via active renal

tubular secretion (e.g., probenecid).

# assessment of safetY

## Data and Safety Monitoring Plan

The proposed study will conform to standard monitoring procedures, and regular review of the study by a Safety Monitoring Committee (SMC). The PIs will ensure appropriate: (1) conduct of the informed consent process (e.g. that informed consent is obtained before proceeding with study procedures); (2) enrollment of study subjects; (3) collection of data and analysis of data; (4) implementation of study procedures to ensure consistent monitoring of subjects; (5) reporting to the SMC, the MRCZ, and the UCSF Committee on Human Research (CHR); and (6) maintenance of the privacy and confidentiality of study subjects. The PIs will be in contact with the research team on a regular basis to review the progress of the study and address any human subject issues that occur. These discussions may involve AE prevention measures, recruiting of appropriate study subjects, research staff training on protection of human subjects and GCP, as well as occurrence of AEs, unexpected incidents, or protocol violations.

## Safety Monitoring Committee

A SMC will be established by the study team to assess at intervals the progress of the pilot study and safety data. The SMC will make recommendations whether to continue, modify or stop the study. The SMC will have written operating procedures and maintain records of all its meetings, including interim results; these will be available for review when the study is complete. The SMC members will not participate in the study as investigators and will not have conflicts of interest regarding the study. The composition of the SMC will include at minimum:

1. A SMC Chair, having experience and expertise in Safer Conception Strategies
2. A scientist with expertise in reproductive health and conception
3. A scientist with expertise in antiretrovirals for HIV prevention

## SMC Process

The first meeting of the SMC will take place prior to the initiation of the pilot study to discuss the protocol, and the Data Safety Monitoring Plan. Meetings will be closed to the public because discussions may address confidential patient data. Meetings may be convened as conference calls as well as in person. An emergency meeting of the SMC may be called at any time should questions of patient safety arise. The SMC may request the presence of study investigators at such meetings. The SMC will determine whether to stop the study for early evidence of safety concerns after a thorough review of interim data. Interim data reports will provide cumulative enrollment figures, and cumulative AEs. Brief clinical descriptions of key events will also be provided. In the event of early study termination, formal reports will be submitted to the IRBs.

## Reports

A data report will be distributed to the SMC at least 10 days prior to a scheduled meeting. This report will generally consist of two parts: 1) information on study progress such as accrual, baseline characteristics, and other general information on study status and 2) any confidential data on study outcomes including adverse event data. A formal report from the SMC will be supplied to the study PIs within 6 weeks of each meeting. Each report should conclude with a recommendation to continue, modify, or to stop the study. This recommendation should be made by formal majority vote. A recommendation to stop the study should be transmitted to the study PIs, UCSF CHR, MRCZ, and NIH as rapidly as possible.

# Eligibility, Enrollment and Procedures for Qualitative interviews (Objective 2)

## Overview

Objective 2 will be informed by the clinical pilot study, and will be conducted after participants exit from the clinical pilot study. While the sample size, eligibility criteria, and analytical framework have been determined (see below), the specific study materials for objective 2, including the interview guides and consent forms, will be developed after the pilot study has started. These materials, and any changes to the sample size, eligibility criteria, and procedures, will be submitted as a modification request to the ethical review committees.

Under Objective 2, we will conduct in-depth semi-structured interviews (IDI) with 20 HIV-serodiscordant couples who participated in the pilot and 10 health care providers in Harare and Chitungwiza (n=5 HIV care providers who participated in the pilot study, 5 primary care providers who did not), in order to characterize provider and patient attitudes and preferences for safer conception strategies, as well as the decision-making process around choosing safer conception strategies. Semi-structured interviews will allow for guidance along relevant themes, but sufficient flexibility to allow for unexpected discoveries of social processes and cultural meanings [^53^](#_ENREF_53).

## Eligibility Criteria

For couples

Inclusion:

1. HIV-discordant couples who participated in the pilot study
2. Willing to participate in one-on-one discussions regarding safer conception strategies.

Exclusion:

1. Unwilling to participate in one-on-one discussions regarding safer conception strategies.

For Health Care Providers (HCP)

Inclusion:

1. HCPs who take care of HIV-affected individuals and couples; up to 5 HCP will have participated in the pilot study
2. Willing to participate in one-on-one discussions regarding safer conception strategies.

Exclusion:

1. Unwilling to participate in one-on-one discussions regarding safer conception strategies.

## Procedures

HIV-discordant couples and providers will be purposively selected. Informed consent will be obtained from each participant individually. The IDI will take place during one study visit at a UZ-UCSF clinic, and will be moderated by a trained research facilitator conducted in the preferred language of the study participants (e.g.Shona). The facilitators will be trained specifically in the delivery of this particular discussion. These interviews are anticipated to take between 1-2 hours. Members of couples will be interviewed separately. Study participants may withdraw from the interview process at any time.

The interviewer will record the audio and will take notes during the IDI. A separate note-taker will attend each session. Participant names and identifiers will be removed from the transcripts to ensure privacy. Interviews will be transcribed in their native language (Shona) by trained research assistants and then translated into English. Validity of the data will be insured with debriefing after the IDIs and review of the final English transcripts for accuracy and back translation of randomly selected transcripts.

# procedures for Cost-effectiveness Study (objective 3)

## Overview

Objective 3 will be informed by the clinical pilot study, and will be conducted after participants have begun enrolling into the clinical pilot study. While the analytical framework has been determined (see below), the specific study materials for objective 3, including the micro-costing and time and motion forms, will be developed after the pilot study has started. These materials, and any other changes to the procedures, will be submitted as a modification request to the ethical review committees.

We will conduct costing studies and develop mathematical models to estimate the cost-effectiveness of different safer conception strategies in this setting. This aim has two objectives: 1) estimate the resources required for providing safer conception options; and 2) estimate the cost-effectiveness of different strategies for safer conception.

## Procedures

For the costing studies, we will observe clinical activities during the pilot study. However, data collection methods will impose no risk or burden on human subjects. We will conduct incremental unit cost analyses using standard micro-costing techniques, which will also include time and motion studies.

### Micro-costing study

Data on costs incurred will be collected for each couple as they go through the each phase of the clinical pilot in order to estimate resources and associated costs required by the UZ-UCSF clinics and patients during implementation of pilot study, e.g. staff, supplies, services, equipment, space, overhead, patient transport. We will collect cost data using standard micro-costing techniques, i.e., an “ingredients based approach”. Specifically, we will calculate costs to identify and screen discordant couples desiring conception, resources required for each component of the study: staff (by type of position), supplies (e.g., medications), services (e.g., publicity), equipment, space, overhead, and patient costs (e.g. transport). We will use standard costing templates from our prior studies of HIV prevention and treatment in sub-Saharan Africa [^54^](#_ENREF_54). They inventory potential resources and assign allocations of shared resources (e.g., staff providing a mix of services) to activity areas and are completed by reviewing clinics’ project expenditure records and by talking with project managers, staff, and where appropriate, participants. Data will be collected from personnel payroll records, equipment/supply purchase records, and other program expenditure databases. We will cost counseling and provision of safer conception strategies separately.

### Time and motion studies

We will conduct time and motion (T&M) studies as needed to provide detail on staff activities. These are structured, precise, direct observations of project activities used to (i) validate micro-costing estimates of time and allocations, and (ii) provide more nuance on task mix. T&M forms will be important to distinguish intervention-specific work from other responsibilities, and to allow us to accurately quantify the staff resources needed for delivery of safer conception strategies. We will determine unit costs for each resource based on actual amounts paid, or if donated or subsidized by using amounts paid by other programs and/or market quotes. We will update unit costs at the time of analysis.

To develop the T&M form, we will generate activity codes based on activities that we expect staff will perform as part of this study. We will then perform a cognitive interview with at least two clinic staff in order to verify that the form covers all activity. Then we will pilot the forms for one day, observing the staff members to ensure that no activities have been overlooked. Each clinic staff member who works on this study will be trained how to use the forms. We will supervise the staff using these forms to ensure they are used correctly. Clinic staff will fill out the T&M forms on an ongoing basis. The forms will cover each staff member’s activities for at least one full week.

# DATA Handling and Record keeping

## Data Collection

Data for Objective 1 will be collected through mobile phone-based and tablet-based structured questionnaires administered to the participants. Questionnaires will be programmed into the tablets and mobile phones. We will use a software platform that allows real-time data collection, transmittal and viewing thus allowing for supervision and immediate implementation of corrective measures to assure data integrity. In addition, we will program complex logic and data mechanisms to further ensure quality of data collected. All data collected with will be sent over a secure cellular data network to the secure server. The server will be compliant with the US Department of Health and Human Services “Health Insurance Portability and Accountability Act” of 1996 (HIPAA) meaning that the participants’ data are considered protected health information and are subjected to a series of administrative, physical, and technical safeguards to assure the confidentiality, integrity, and availability of the electronic data being transmitted. Data will be viewable online in easy-to-read reports and can be downloaded by authorized users (in this case the P.I and Co-P.I or designee) into Excel files for further analysis.

Clinical data and laboratory data will be captured on source documents and will be entered into the database.

Data for Objective 2 (qualitative interviews) will be audio-recorded and transcribed and translated and kept in locked filing cabinets.

Data for Objective 3 (costing data) will be collected in Microsoft Excel and imported to a database for storage and manipulation. Although there is no confidential patient information in the cost data, it will be integrated with standard secure methods used for other data in the study.

## Data Storage

Study record keeping and access to identifying information will be guided by UCSF and MRCZ ethical guidelines. All records will be kept confidential. Participants will be identified primarily by their study number and patient names will not be entered into the computerized database. All hard-copy data such as informed consent forms will be stored in a secured filing cabinet in a locked, access-limited room at the study clinic. Additional records will be kept in the clinical and laboratory record books, which will be stored in the local study laboratory. All soft copy data such as the data collected via mobile phones or tablets will be stored in password-protected files in access-controlled computers. No individual identities will be used in any reports or publications resulting from the study. Care will be taken to maintain confidentiality of all study data. Only the study coordinator and study investigators will have access to the data.

## Data Management

All data will be collected and stored in locked cabinets at the study site. Data entry will be conducted on site, and stored in password-protected databases on a secure computer with access limited to study staff. The data files will be regularly backed up on secure servers. Data will be analyzed as described in *Section 11. Statistical considerations*.

## Types of Data

Objective 1 Pilot Study: Data from interviews and laboratory results will be entered into a password protected database.

Objective 2 Qualitative Interviews: In-depth interviews will be audio-recorded and transcribed and translated and kept in locked filing cabinets.

Objective 3: Costing data: Cost data will be collected in Microsoft Excel and imported to a database for storage and manipulation. Although there is no confidential patient information in the cost data, it will be integrated with standard secure methods used for other data in the study.

## Study Records Retention

## Research records will be maintained for at least 3 years following the completion of the research and finalization of publications.

# Statistical considerations

## *Analysis Plan*

**Objective 1:** Descriptive summaries of the proportion of participants measuring uptake of different strategies, adherence, retention in care, sexual behaviors, condom use, patient satisfaction, HIV and pregnancy incidence, obstetric and neonatal outcomes, and adverse events will be presented and accompanied by 95% confidence intervals. These estimates will also be stratified by characteristics of interest (e.g. sex, age, method of safer conception, etc.).

**Objective 2:** Recordings of interviews will be transcribed in their native language (Shona) by trained research assistants and imported into Transana, which facilitates secure, remote, collaborative analysis of notes and transcripts synchronized to the source audio [^55^](#_ENREF_55). All interviews will be translated into English for further analysis using ATLAS.ti, which facilitates the sorting, organizing, and relating of coded segments of textual data in accordance with principles of grounded theory analysis. The investigative team will iteratively analyse the interviews and field notes, discuss and resolve discrepancies regarding data segments that are particularly rich or difficult to code, and develop a common set of codes describing patterns observed in the data. The presence of individual interviews from both members of the couple will provide a rich dataset that will illustrate how discrepancies and similarities in attitudes within couples impact their decision-making and preferences towards different safer conception strategies. These methods have been used in prior couples-based studies to inform the development of interventions [^56^](#_ENREF_56).

**Objective 3:**

Microcosting and T&M studies: During the pilot cohort study, we will quantify the resources required for delivered services and determine unit costs for each resource based on actual amounts paid, or if donated or subsidized, by using amounts paid by other programs and/or market quotes (i.e., economic costs). We will update unit costs at the time of final analysis. We will calculate the cost of the different strategies for safer conception alone and in combination per month per couple. Total cost will reflect the sum of [# of resources * unit cost] (measured as described above). In order to determine net costs, we will estimate the costs of induced health services, such as ART post-partum. These health services unit costs will be drawn from published studies and other sources, such as ART price inventories and the WHO-CHOICE unit cost database [^57^](#_ENREF_57). Induced costs will also reflect the savings associated with averted HIV infections, as well as differences in HIV treatment costs associated with altered disease course, due to reductions in morbidity and evolving use of ART regimens. Future costs and health outcomes will be discounted at 3% (up to 10% in sensitivity analyses).

Cost-effectiveness models: The cost-effectiveness ratio will be calculated as the [net program cost] divided by the [number of HIV infections averted], or the [number of associated DALYs averted]. The net program cost will reflect savings due to avoided lifetime HIV medical costs, and more generally for induced cost effects. We will also estimate averted secondary transmission of HIV infections (both horizontal and vertical), i.e., infections not occurring due to partners staying HIV-negative.

For this analysis, we will use simple deterministic transmission models that incorporate number of partners and their HIV prevalence, coital frequency, condom use, and risk per sex act. These models will estimate the cost-effectiveness of strategies for safer conception. We will simulate the use of different strategies for safer conception alone and in combination, and quantify the effects on HIV transmission, life years saved, and cost per infected averted. The model for discordant couple transmission will include risk factors characterizing composition of couples (e.g., age, sex of HIV positive partner, current CD4 count/viral load), condom use, conception and pregnancies, variations in coital frequency, and contact with other potentially infected partners. In order to determine the best overall strategies for safer conception among HIV discordant couples, the impact and cost of the full range of possible different strategies will be calculated. We will conduct sensitivity analyses to assess the effect of uncertainty in input values, such as adherence and costs on outcomes.

# Ethical considerations

## Ethical Considerations

Currently, HIV discordant couples who attempt to conceive place themselves at risk of HIV transmission. Provision of safer conception in this context aims to help promote couples’ right to conceive while at the same time decreasing the risk of HIV transmission. It has been argued that it would be unethical to withhold proven HIV prevention strategies to discordant couples that are trying to conceive.[^21^](#_ENREF_21)

## Institutional Review Board

The protocol, informed consent form(s), recruitment materials and all participant materials will be submitted to the UCSF Committee on Human Research (CHR) and the Medical Research Council of Zimbabwe (MRCZ) for approval. Approval of both the protocol and the consent form must be obtained before any participant is enrolled. The Joint Research Ethics Committee of Parirenyatwa Hospital and the University Of Zimbabwe College Of Health Sciences will review the proposal and once approved, will be submitted to MRCZ; the Medicines Control Authority of Zimbabwe (MCAZ) will then review and approve study drug, followed by final review and approval by Research Council of Zimbabwe (RCZ). Any amendment to the protocol will require review and approval by all IRBs before the changes are implemented in the study.

## Process of Obtaining Informed Consent and Documentation of Consent

The study team has experience in obtaining informed consent from individuals and couples for research and clinical trials within Zimbabwe. The informed consent procedure for this study has been designed to maximize understanding of potential risks. All consent forms will be translated into the local languages (Shona) and back-translated into English to ensure correct use of language. Consent forms will be read aloud to each participant individually by study interviewers. After reading the consent forms, prior to seeking a signature, interviewers will ask participants to summarize the study and explain the reasons why they want to participate. A comprehension questionnaire will be administered to determine if the study objectives, procedures and potential risks and benefits are understood by the subject. If there are cultural, literacy or political reasons why signature is not appropriate, individuals will be allowed to mark consent forms with a thumbprint (thumb printing is standard for clinical research where individuals do not write), along with a co-signature by a witness otherwise not affiliated with the study. At this point, any misunderstandings regarding procedures, risks, or benefits can be clarified. Individuals will be provided with information on how to contact the study staff to report AEs. Study staff will be trained in the need to assure that individuals provide voluntary informed consent. They may withdraw consent at any time throughout the course of the study. A copy of the signed informed consent document will be given to participants for their records. The rights and welfare of the participants will be protected by emphasizing to them that the quality of their clinical care will not be adversely affected if they decline to participate in this study. The consent process will be documented in the clinical or research record.

## Participant Confidentiality

Successful implementation of the study will require that all study staff strictly maintain the confidentiality of all study participants. Participants will be assigned a unique identification number for all data collected over the course of the study. All data and information collected will be kept in locked file cabinets in an access-limited room at our research facility. All project staff will be trained on procedures for maintaining confidentiality and asked to sign a pledge of confidentiality. As this is a study involving couples, additional measures are needed to protect each participant’s confidentiality from their partner. From our prior work, we have developed procedures to minimize risk. Couples will arrive together, but be consented and interviewed separately. Prior to their separation, study staff will inform them that they may contact the staff member who interviewed them, but they will not be permitted to have contact with the interviewer who consented or administered the questionnaire to their partner. This procedure will enhance the participants’ confidence that the information they disclose will be kept confidential and not disclosed to their partner.

## Potential Benefits, Risks and Plans for Mitigating Those Risks

### Potential Benefits

**Potential Benefit to Individual Participants.** The potential benefit of participating in this research is the opportunity to receive options for safer conception, including ART, PrEP, instruction on artificial vaginal insemination, semen washing with IUI, fertility monitoring, syndromic management for STIs, screening for cervical cancer, provision of folic acid supplementation, and condoms, and PMTCT by an experienced and attentive staff. Study participants may learn about safer methods of conception that can prevent transmission of HIV infection within their partnership, and experience improved referral to resources to assist them and their family members in coping with HIV infection and other issues. Participants will also receive counseling sessions that will emphasize HIV risk reduction, adherence, and pre-conception and perinatal care. Participants will be provided with referrals for additional counseling and services if needed for disclosure, partner violence, alcohol, or substance abuse. Through our prior work in the community, we have compiled a list of community-based resources for couples, including general health services, mental health counseling, partner violence, substance use, and other issues. These lists will continue to be updated, and will systematically be provided to every couple.

**Social Benefit.** The pilot study will provide us with critical data on uptake of different strategies, adherence, retention in care, change in sexual behavior in the context of safer conception, preliminary estimates of pregnancy incidence, and obstetric and neonatal outcomes. This data will be used to inform the cost-effectiveness models. The results of this program of research may inform the scale up of safer conception programs in Zimbabwe, and may eventually be useful to other sub-Saharan African settings. It should also be noted that developing countries with generalized HIV epidemics would receive the most direct benefit of the research in contrast to the US or Europe where other options to reduce sexual transmission among HIV-discordant couples desiring conception are available. Thus, this proposal also responds to the ethical principle of justice; the group experiencing the research risk receives the benefit of the research.

### Potential Risks and Plans for Mitigating Those Risks

**a. Loss of privacy.** Participation in research may involve loss of privacy of research subjects. Personal and medical information will be collected from participants and only study staff will have access to this information.

**Plans to mitigate:** Every effort will be made to keep subjects participation in the trial confidential. All project staff will be trained on procedures for maintaining confidentiality and asked to sign a pledge of confidentiality. Study staff will be trained to avoid answering any questions posed by any participants’ undisclosed family or friends. All home visits will be done in unmarked vehicles and study staff will not disclose their affiliation without the permission of the study participant.

**b. Embarrassment or anxiety.** Participants may become embarrassed, worried, or anxious when talking about their perspectives on safer conception for HIV-affected couples.

**Plans to mitigate:** All project staff have been trained in the protection of human subjects. We will use trained interviewers to minimize potential embarrassment.

**c. HIV testing.** Being tested for HIV may cause anxiety or stress regardless of the test results. Receiving a positive test result for oneself or a child may make subjects very upset.

**Plans to mitigate:** We will provide referrals for additional counseling and services if needed for disclosure. Through our prior work in the community, we have compiled a list of community-based resources for couples, including general health services, mental health counseling, and other issues.

**d. Drawing blood by venipuncture or finger-stick.** Blood draws may cause momentary discomfort and residual bruising. Rarely, blood collection may cause fainting or infection.

**Plans to mitigate:** Only trained medical staff will collect these samples to mitigate discomfort and complications.

**e. Stigma regarding HIV infection.** Although every effort will be made to keep subjects and couples participation in the trial confidential, there is the possibility that others may inadvertently learn of subjects’ participation in the study or that a subject is taking HIV medications and stigmatization of their condition might result.

**Plans to mitigate:** Every effort will be made to keep subjects and couples participation in the trial confidential. Couples will receive counseling aimed at stigma reduction as needed.

**f. Risk of ART and PrEP use in men and women in this study.** ART regimens will be consistent with the current WHO and Zimbabwean Guidelines for Antiretroviral Therapy for the Prevention and Treatment of HIV (NMTPAC/MOHCC).

The Partners PrEP Study demonstrated that PrEP (including Truvada) was safe for use in heterosexual men and women from Africa. There were no statistically significant differences in the frequency of deaths, serious adverse events, adverse events overall, or key laboratory adverse events (specifically, creatinine elevation and phosphorus decrease) for those receiving PrEP compared to those receiving placebo.

Participants are at risk of developing drug resistance as a result of poor adherence to their ART regimen. This would affect the available options for treatment in the future when their immune function declines to the point that ART is required for their health.

**Plans to mitigate:** All potential participants will be counseled at length about this possibility at the enrollment visits, and adherence assessments and counseling will continue throughout the study.

If HIV-uninfected women or men in serodiscordant couples choose to use PrEP, the couples will be educated about the potential risks and benefits. Laboratory testing for HIV infection, baseline renal function will be performed before initiating PrEP. Individuals receiving PrEP will also be monitored for potential side effects such as renal dysfunction and clinical toxicities. They will be educated about symptoms associated with acute HIV infection and advised to contact the study site immediately for further evaluation should symptoms occur. HIV-uninfected partners will undergo regular HIV testing to detect HIV infection quickly. Should HIV infection be documented, PrEP will be discontinued to minimize selection of drug-resistant virus, and measures will be instituted to prevent perinatal transmission if pregnancy occurs.

Decisions to hold PrEP or ART due to clinical and/or other laboratory safety reasons, or in the event of overdose, will be at the discretion of site Investigator, in consultation with the PIs.

Clinical symptoms will be systematically assessed in a structured medical history administered to participants taking ART or PrEP. Clinical side effects of FTC/TDF (Truvada) that have been reported are primarily gastrointestinal, including nausea, vomiting, and flatulence.

**g. Maternal and fetal risks associated with ART and PrEP use in pregnancy.** Pregnant women will receive ART in accordance with the most current Zimbabwean Guidelines for Antiretroviral Therapy for the Prevention and Treatment of HIV (NMTPAC/MOHCC). These drugs have all been tested in preclinical studies involving gravid animals and clinical studies involving non-pregnant and pregnant women. The antiretroviral medications are all considered safe and are first-line regimens per the Panel on Treatment of HIV-Infected Pregnant Women and Prevention of Perinatal Transmission [^18^](#_ENREF_18).

While there are limited data on the safety of FTC/TDF (Truvada) in pregnant and breastfeeding women, the available human and animal data suggest that Truvada does not increase the risk of major birth defects overall compared to the background rate. Because the studies in humans cannot rule out the possibility of harm, Truvada should be used during pregnancy only if clearly needed. Pregnancy is a high-risk period for HIV acquisition and transmission.

**Plans to mitigate:** We will follow current clinical guidelines for use of ART in pregnancy to minimize maternal and fetal risk (NMTPAC/MOHCC). The benefit of conceiving a child with low risk of vertical transmission potentially outweighs the risk posed to the pregnant women and fetuses in this study. Children born to HIV infected mothers will be treated according to Zimbabwean National Guidelines.

We will follow the same procedures that we have followed in other PrEP studies of FTC/TDF (Truvada) at the study site: women will be counseled on the available safety information related to use of FTC/TDF (Truvada) in pregnancy, and monitored monthly for pregnancy. After conception, women will be counseled about the risks and benefits of FTC/TDF (Truvada), taking into account the potential increased risk of HIV acquisition during pregnancy, and will be given the choice to continue FTC/TDF (Truvada) through pregnancy. Study staff will be trained to provide participants with a summary of current understanding of the literature about risks and benefits of PrEP in pregnancy. For this study, FTC/TDF (Truvada) will be discontinued in HIV-negative women prior to breastfeeding. HIV-1 uninfected women will be encouraged to breastfeed their infants according to WHO and national guidelines.

**h. Coercion to participate.** When conducting research with couples there exists the possibility that participating in the study could result in coercion of one partner by another to participate.

**Plans to mitigate:** Although we have not had problems in the past in our couples’ studies in Harare with individuals reporting being there under coercion, we will institute procedures and train staff to minimize this risk and manage cases of suspected coercion based on procedures in place for our other couples’ studies. Staff members will be trained to be sensitive to the possibility that one member of the couple was pressured or coerced by the other partner to participate in the study. During the consenting process, and follow-up visits, questions may be posed such as “Did you come here freely?” or “Will something bad happen to you if you say no?”. We will institute procedures and train staff to minimize this risk.

**i. Exposure to HIV.** Participants who are using condoms to prevent HIV transmission may be at high risk of HIV transmission if a condom breaks during intercourse.

**Plans to mitigate**: Condoms will be provided to study participants, and participants will be trained on the correct use of condoms. Couples who report condom failure will be offered post-exposure prophylaxis according to current Guidelines for Antiretroviral Therapy for the Prevention and Treatment of HIV in Zimbabwe (NMTPAC/MOHCC).

### Alternatives to Participation

Safer conception counseling and strategies are not accessible to most HIV-discordant couples in sub-Saharan Africa. In the absence of any intervention, couples may try natural intercourse to conceive. Discordant-couples who try natural intercourse to conceive are potentially at higher risk of HIV transmission.

Participants will be told that they are free to not take part in this study if they do not want to. Medical care and health examinations are available at the health centers for all. The participant will be told that they may stop taking part in this study at any time, and that refusing to take part will not affect their current or future medical care in any way.

### Provisions for the Protection of Privacy

Participants will be assigned a unique identification number for all data collected. Consent, study interviews and in-depth interviews (IDI) will be conducted in private rooms. All data and information collected (IDI transcripts, questionnaires, consent forms, lab results) will be kept in locked file cabinets in an access-limited room at the study site. All project staff will be trained on procedures for maintaining confidentiality and asked to sign a pledge of confidentiality. All electronic data will be protected by password.

### Measures for Ensuring Confidentiality of Data (access to data, duration and location of storage of data)

Successful implementation of the study will require that all study staff strictly maintain the confidentiality of all study participants. Confidentiality will be protected by using unique identifiers, rather than proper names, for the purpose of all data collection, analysis, and storage. Names and identifiers will not be used in any presentations or publications. All data and project forms will be coded only with the unique identifiers. All interviews recordings (digital audio files) will be stored as encrypted files marked only with a unique numeric identifier. All data and information collected will be kept in locked file cabinets in an access-limited room at our study site. All electronic data will be protected by password. All project staff will be trained on procedures for maintaining confidentiality and asked to sign a pledge of confidentiality. Research records will be maintained for at least 3 years following the completion of the research.

### Plans for Disseminating Study Findings to Study Participants

Following the completion of the study and analysis of the data, the study team will offer to share an aggregate summary of the study findings with the study participants, and answer participant’s questions about the study findings and implications.

# Quality assurance

An internal quality assurance/quality control system will be put in place to ensure the integrity of the study, including that all regulatory processes are followed.

# expected application of results

While evidence-based recommendations for safer conception have recently been developed for settings in sub-Saharan Africa, including Zimbabwe, to date no studies have been done in Zimbabwe or elsewhere to evaluate the actual (rather than hypothetical) feasibility of and preferences for these strategies among providers and patients. Moreover, no studies have been done to evaluate the potential cost-effectiveness of the different strategies for safer conception alone or in combination on HIV prevention. This project will address this critical gap in HIV prevention. Results from this study will inform safer conception strategies in Zimbabwe, and may serve as a model for other countries that want to implement safer conception strategies and estimate their cost-effectiveness in their settings. Improving reproductive health is a public priority in Zimbabwe. Provision of safer conception strategies to HIV affected couples who desire conception will not only help couples achieve their reproductive rights, but may simultaneously help Zimbabwe to end the AIDS epidemic.

# Literature References

1. Cooper D, Harries J, Myer L, Orner P, Bracken H, Zweigenthal V. "Life is still going on": reproductive intentions among HIV-positive women and men in South Africa. *Soc Sci Med* 2007; **65**(2): 274-83.

2. Smith DJ, Mbakwem BC. Life projects and therapeutic itineraries: marriage, fertility, and antiretroviral therapy in Nigeria. *AIDS* 2007; **21 Suppl 5**: S37-41.

3. Nattabi B, Li J, Thompson SC, Orach CG, Earnest J. A systematic review of factors influencing fertility desires and intentions among people living with HIV/AIDS: implications for policy and service delivery. *AIDS Behav* 2009; **13**(5): 949-68.

4. Hageman K VC, Haworth A, Henderson F, Kancheya N, Zulu I, Lakhi S, Chomba E, Tichacek A, Allen S. Condom use among HIV discordant couples in Zambia: barriers to consistency Abstract WEPEC028. *4th Annual International AIDS Society Conference* 2007.

5. Bekker L-G, Black V, Myer L, et al. Guideline on Safer Conception in Fertile HIV-Infected Individuals and Couples. *The South African Journal of HIV Medicine* 2011: 31-44.

6. Ministry of Health, Kenya; National AIDS and STI Control Programme (NASCOP). Guidelines on Use of Antiretroviral Drugs for Treating and Preventing HIV Infection: A rapid advice, 2014. June 2014.

7. National AIDS and STI Control Programme MoH, Kenya. Kenya AIDS Indicator Survey 2012: Preliminary Report. . Nairobi, Kenya., September 2013.

8. National AIDS and STI Control Programme MoH K. Kenya AIDS Indicator Survey 2007: Preliminary Report. 2008.

9. Chemaitelly H, Cremin I, Shelton J, Hallett TB, Abu-Raddad LJ. Distinct HIV discordancy patterns by epidemic size in stable sexual partnerships in sub-Saharan Africa. *Sex Transm Infect* 2012; **88**(1): 51-7.

10. Guthrie BL, de Bruyn G, Farquhar C. HIV-1-discordant couples in sub-Saharan Africa: explanations and implications for high rates of discordancy. *Curr HIV Res* 2007; **5**(4): 416-29.

11. Lingappa JR, Lambdin B, Bukusi EA, et al. Regional differences in prevalence of HIV-1 discordance in Africa and enrollment of HIV-1 discordant couples into an HIV-1 prevention trial. *PLoS One* 2008; **3**(1): e1411.

12. Allen S, Chomba E, Karita E, Kilembe W, Inambao M, Streeb G. The contribution of HIV-discordant relationships to new HIV infections: a rebuttal. *AIDS* 2011; **25(10)**: 1341-3.

13. Ministry of Health U. Uganda HIV/AIDS Sero-Behavioural Survey 2004-2005. *MOH and ORC Macro* 2006.

14. Dunkle KL, Stephenson R, Karita E, et al. New heterosexually transmitted HIV infections in married or cohabiting couples in urban Zambia and Rwanda: an analysis of survey and clinical data. *Lancet* 2008; **371**(9631): 2183-91.

15. Coburn BJ, Gerberry DJ, Blower S. Quantification of the role of discordant couples in driving incidence of HIV in sub-Saharan Africa. *Lancet Infect Dis* 2011; **11**(4): 263-4.

16. Brubaker S, Bukusi E, Odoyo J, Achando J, Okumu A, Cohen C. Pregnancy and HIV transmission among HIV discordant couples in a clinical trial in Kisumu, Kenya. *HIV Med* 2011; **12(5)**: 316-21.

17. Rispel LC, Metcalf CA, Moody K, Cloete A, Caswell G. Sexual relations and childbearing decisions of HIV-discordant couples: an exploratory study in South Africa and Tanzania. *Reprod Health Matters* 2011; **19**(37): 184-93.

18. Panel on Treatment of HIV-Infected Pregnant Women and Prevention of Perinatal Transmission. Recommendations for Use of Antiretroviral Drugs in Pregnant HIV-1-Infected Women for Maternal Health and Interventions to Reduce Perinatal HIV Transmission in the United States. *Available at:* <http://aidsinfonihgov/ContentFiles/PerinatalGLpdf> May 24, 2010: 1-117.

19. Anglemyer A, Rutherford GW, Baggaley RC, Egger M, Siegfried N. Antiretroviral therapy for prevention of HIV transmission in HIV-discordant couples. . *Cochrane Database of Systematic Reviews* 2011; (Issue 5): No.: CD009153. DOI: 10.1002/14651858.CD009153.

20. Cohen MS, Chen YQ, McCauley M, et al. Prevention of HIV-1 Infection with Early Antiretroviral Therapy. *NEJM* 2011; **Epub July 18, 2011**.

21. Mantell JE, Smit JA, Stein ZA. The right to choose parenthood among HIV-infected women and men. *Journal of public health policy* 2009; **30**(4): 367-78.

22. Baeten JM, Donnell D, Ndase P, et al. Antiretroviral prophylaxis for HIV prevention in heterosexual men and women. *The New England journal of medicine* 2012; **367**(5): 399-410.

23. Barreiro P, del Romero J, Leal M H, V, Asencio R, de Mendoza C, et al. Natural pregnancies in HIV-serodiscordant couples receiving successful antiretroviral therapy. *J Acquir Immune Defic Syndr* 2006; **43**: 324-6.

24. Vernazza PL, Graf I, Sonnenberg-Schwan U, Geit M, Meurer A. Preexposure prophylaxis and timed intercourse for HIV-discordant couples willing to conceive a child. *AIDS* 2011; **25**(16): 2005-8.

25. Mastro TD, Cohen MS, Rees H. Antiretrovirals for safer conception for HIV-negative women and their HIV-1-infected male partners: how safe and how available? *AIDS* 2011; **25**(16): 2049-51.

26. Asamoah-Odei E, Garcia Calleja JM, Boerma JT. HIV prevalence and trends in sub-Saharan Africa: no decline and large subregional differences. *Lancet* 2004; **364**(9428): 35-40.

27. Kenya National Bureau of Statistics (KNBS) and ICF Macro. 2010. Kenya Demographic and HealthSurvey 2008-09. Calverton MKaIM.

28. Fakoya A, Lamba H, Mackie N, et al. British HIV Association, BASHH and FSRH guidelines for the management of the sexual and reproductive health of people living with HIV infection 2008. *HIV Med* 2008; **9**(9): 681-720.

29. Centers for Disease C, Prevention. Revised guidelines for HIV counseling, testing, and referral. *MMWR Recommendations and reports : Morbidity and mortality weekly report Recommendations and reports / Centers for Disease Control* 2001; **50**(RR-19): 1-57; quiz CE1-19a1-CE6-a1.

30. Global Health Initiative Kenya Strategy 2011-2014. USAID. Revision: January 18, 2011.

31. Dworkin SL. Sample size policy for qualitative studies using in-depth interviews. *Archives of sexual behavior* 2012; **41**(6): 1319-20.

32. Allison EH, Seeley JA. HIV and AIDS among fisherfolk: a threat to ‘responsible fisheries’? *Fish and Fisheries* 2004.

33. Gray A, Smit J, Manzini N, Beksinska M. Systematic review of contraceptive medicines. Does choice make a difference? . *University of Witwatersrand, Johannesberg* 2006.

34. Ethics Committee of the American Society for Reproductive Medicine. Human immunodeficiency virus and infertility treatment. Fertil Steril. Jun 2010;94(1):11-15. Available at <http://www.ncbi.nlm.nih.gov/pubmed/20236636>.

35. Baeten J, Celum C, al. e, Team. TPPS. Antiretroviral pre-exposure prophylaxis for HIV-1 prevention among heterosexual African men and women: the Partners PrEP Study. [Abstract MOAX01.]. 6th IAS Conference on HIV Pathogenesis, Treatment and Prevention July 17-20, 2011; Rome, Italy; 2011.

36. Mmeje O, Cohen CR, Cohan D. Evaluating safer conception options for HIV-serodiscordant couples (HIV-infected female/HIV-uninfected male): a closer look at vaginal insemination. *Infectious diseases in obstetrics and gynecology* 2012; **2012**: 587651.

37. Lynch CD, Jackson LW, Buck Louis GM. Estimation of the day-specific probabilities of conception: current state of the knowledge and the relevance for epidemiological research. *Paediatric and perinatal epidemiology* 2006; **20 Suppl 1**: 3-12.

38. Zinaman MJ. Using cervical mucus and other easily observed biomarkers to identify ovulation in prospective pregnancy trials. *Paediatric and perinatal epidemiology* 2006; **20 Suppl 1**: 26-9.

39. Scarpa B, Dunson DB, Giacchi E. Bayesian selection of optimal rules for timing intercourse to conceive by using calendar and mucus. *Fertility and sterility* 2007; **88**(4): 915-24.

40. <http://www.who.int/reproductivehealth/topics/infertility/definitions/en/>. <http://www.who.int/reproductivehealth/topics/infertility/definitions/en/>.

41. Chomba E, Allen S, Kanweka W, et al. Evolution of couples' voluntary counseling and testing for HIV in Lusaka, Zambia. *J Acquir Immune Defic Syndr* 2008; **47**(1): 108-15.

42. Horne R, Weinman J, Hankins M. The Beliefs about Medicines Questionnaire: The development and evaluation of a new method for assessing the cognitive representation of medication. *Psychology & Health* 1999 **14(1)**: 1-24.

43. Kraft J, Harvey S, Thorburn S, Henderson J, Posner S, Galavotti C. Intervening with couples: assessing contraceptive outcomes in a randomized pregnancy and HIV/STD risk reduction intervention trial. *Womens Health Issues* Jan - Feb 2007; **17(1)** 52-60.

44. Kurdek L. The deterioration of relationship quality for gay and lesbian cohabiting couples: A five-year prospective longitudinal study. *Personal Relationships* Dec 1996; **3(4)**: 417-42.

45. Pulerwitz J, Gortmaker SL, DeJong W. Measuring sexual relationship power in HIV/STD research. *Sex Roles* Apr 2000; **42(7-8)**: 637-60.

46. Turan JM, Bukusi EA, Onono M, Holzemer WL, Miller S, Cohen CR. HIV/AIDS Stigma and Refusal of HIV Testing Among Pregnant Women in Rural Kenya: Results from the MAMAS Study. *AIDS Behav* 2011; **15**(6): 1111-20.

47. Byakika-Tusiime J, Crane J, Oyugi JH, et al. Longitudinal antiretroviral adherence in HIV+ Ugandan parents and their children initiating HAART in the MTCT-Plus family treatment model: role of depression in declining adherence over time. *AIDS Behav* 2009; **13 Suppl 1**: 82-91.

48. Maggiolo F, Ravasio L, Ripamonti D, et al. Similar adherence rates favor different virologic outcomes for patients treated with nonnucleoside analogues or protease inhibitors. *Clin Infect Dis* 2005; **40**(1): 158-63.

49. Mills EJ, Nachega JB, Buchan I, et al. Adherence to antiretroviral therapy in sub-Saharan Africa and North America: a meta-analysis. *JAMA* 2006; **296**(6): 679-90.

50. Weiser S, Wolfe W, Bangsberg D, et al. Barriers to antiretroviral adherence for patients living with HIV infection and AIDS in Botswana. *J Acquir Immune Defic Syndr* 2003; **34**(3): 281-8.

51. Nachega JB, Stein DM, Lehman DA, et al. Adherence to antiretroviral therapy in HIV-infected adults in Soweto, South Africa. *AIDS Res Hum Retroviruses* 2004; **20**(10): 1053-6.

52. Council TP. Adapting Focused Antenatal Care: Lessons from Three African Countries. *Program Brief No 11* 2008; **Available at:** <http://www.popcouncil.org/pdfs/frontiers/pbriefs/PB11.pdf>.

53. Bernard HR, editor. Research Methods in Anthropology: Qualitative and Quantitative Approaches. . Thousand Oaks, CA: Sage; 1994.

54. Marseille E, Dandona L, Marshall N, et al. HIV prevention costs and program scale: data from the PANCEA project in five low and middle-income countries. *BMC Health Serv Res* 2007; **7**: 108.

55. Woods D, Fassnacht C. Transana Software. 2010, The Board of Regents of the University of Wisconsin: Madison, WI.

56. Leddy A, Johnson MO, Dladla S, Darbes LA. South African couples’ preferences for the delivery of a couples-based voluntary counseling and testing (CBVCT) intervention. Poster presented at the XIX International AIDS Conference, Washington, D.C. 2012.

57. <http://www.who.int/choice/costs/en/>.

58. Semprini AE, Levi-Setti P, Bozzo M, et. al. Insemination of HIV-negative women with processed semen of HIV-positive partners. *Lancet* 1992; Nov 28; **340(8831)**: 1317-9.

59. Zafer M, Horvath H, Mmeje O, et. al. Effectiveness of semen washing to prevent human immunodeficiency virus (HIV) transmission and assist pregnancy in HIV-discordant couples: a systematic review. *Fertility and Sterility* 2016. March;**105(3)**: 645-55.

60. https://www.eshre.eu/

APPENDIX A: Schedule of Events - Screening, Enrollment/Run-In, and Delivery of Strategies

|  |  | **2 month run-in** | | | **Up to 12 months of pregnancy attempts and follow-up** | | | | | | | | | | | |
| --- | --- | --- | --- | --- | --- | --- | --- | --- | --- | --- | --- | --- | --- | --- | --- | --- |
| **General Procedures** | **Screening** | **Enrollment** | **M** | **M** | **M** | **M** | **M** | **M** | **M** | **M** | **M** | **M** | **M** | **M** | **M** | **M** |
|  |  | **M0** | **1** | **2** | **3** | **4** | **5** | **6** | **7** | **8** | **9** | **10** | **11** | **12** | **13** | **14** |
| Obtain informed consent | B | B |  |  | HIV-negative women using PrEP who become pregnant will be counseled and will provide informed consent to continue use of PrEP during pregnancy | | | | | | | | | | | |
| Determine/confirm eligibility | B | B |  |  |  |  |  |  |  |  |  |  |  |  |  |  |
| Collect demographic information | B |  |  |  |  |  |  |  |  |  |  |  |  |  |  |  |
| Collect/update locator information | B | B |  |  | Quarterly for all participants through the end of study follow-up | | | | | | | | | | | |
|  |  | **2 month run-in** | | | **Up to 12 months of pregnancy attempts and follow-up** | | | | | | | | | | | |
| **Questionnaires** | **Screening** | **Enrollment M0** | **M**  **1** | **M**  **2** | **M**  **3** | **M**  **4** | **M**  **5** | **M**  **6** | **M**  **7** | **M**  **8** | **M**  **9** | **M**  **10** | **M**  **11** | **M**  **12** | **M**  **13** | **M**  **14** |
| Baseline Behavioral Screening/ Eligibility | B |  |  |  |  |  |  |  |  |  |  |  |  |  |  |  |
| Baseline Socio-demographics, pregnancy intention, HIV testing, and Sexual History | B |  |  |  |  |  |  |  |  |  |  |  |  |  |  |  |
| Screening/ Enrollment Eligibility | B | B |  |  |  |  |  |  |  |  |  |  |  |  |  |  |
| Baseline Medical history | B | B |  |  |  |  |  |  |  |  |  |  |  |  |  |  |
| Baseline Questionnaire on Beliefs about methods of safer conception, relationship power, and HIV-related stigma |  | B |  |  | Repeated at month 6 or at conception(whichever comes first), and at last study visit | | | | | | | | | | | |
| Targeted Clinical Questionnaire & AEs |  | B | B | B | Monthly in all participants until conception occurs; quarterly thereafter, and at last study visit | | | | | | | | | | | |
| Baseline and Monthly Follow-up Menstrual and Pregnancy Form |  | A♀ | A♀ | A♀ | Monthly in A♀ until conception occurs | | | | | | | | | | | |
| Monthly Follow-Up Questionnaire on Patient Satisfaction With Safer Conception Method |  |  | B | B | Monthly in all participants until conception occurs; quarterly thereafter, and at last study visit | | | | | | | | | | | |
| Monthly follow-up Adherence Questionnaire on Safer Conception Strategies, Sexual Behavior, and Condom Use |  |  | B | B | Monthly in all participants until conception occurs; quarterly thereafter in those using ART or PrEP during pregnancy, and at last study visit | | | | | | | | | | | |
|  |  | **2 month run-in** | | | **Up to 12 months of pregnancy attempts and follow-up** | | | | | | | | | | | |
| **Counseling** | **Screening** | **Enrollment M0** | **M**  **1** | **M**  **2** | **M**  **3** | **M**  **4** | **M**  **5** | **M**  **6** | **M**  **7** | **M**  **8** | **M**  **9** | **M**  **10** | **M**  **11** | **M**  **12** | **M**  **13** | **M**  **14** |
| HIV pre/posttest counseling | B | B | HIV- | HIV- | Monthly in the HIV- partner until conception occurs; quarterly thereafter, and at study exit | | | | | | | | | | | |
| Nutritional counseling |  | B |  |  |  |  |  |  |  |  |  |  |  |  |  |  |
| Intensive counseling to enhance HIV prevention to partner and baby |  | B | B | B | Quarterly for all participants through end of pregnancy | | | | | | | | | | | |
| Adherence counseling |  | B | B | B | Quarterly for all participants through conception. As needed when low adherence is reported ¥ | | | | | | | | | | | |
| Fertile days counseling |  | B | B | B | Quarterly for all participants through conception | | | | | | | | | | | |

|  | |  | **2 month run-in** | | | | **Up to 12 months of pregnancy attempts and follow-up** | | | | | | | | | | | |
| --- | --- | --- | --- | --- | --- | --- | --- | --- | --- | --- | --- | --- | --- | --- | --- | --- | --- | --- |
| **Clinical Procedures** | **Screening** | | **Enrollment**  **M0** | | **M**  **1** | **M**  **2** | **M**  **3** | **M**  **4** | **M**  **5** | **M**  **6** | **M**  **7** | **M**  **8** | **M**  **9** | **M**  **10** | **M**  **11** | **M**  **12** | **M**  **13** | **M**  **14** |
| Anemia screening | B | |  | |  |  |  |  |  |  |  |  |  |  |  |  |  |  |
| Cervical cancer screening | A♀ | |  | |  |  | Annually in HIV+ women; 12 week post-partum visit if pregnant  (per Zimbabwean NMTPAC/MOHCC guidelines) | | | | | | | | | | | |
| Collect medical history | B | | B | |  |  |  |  |  |  |  |  |  |  |  |  |  |  |
| Perform targeted physical exam and symptom screening | B | | B | |  |  | Quarterly in both participants until conception occurs | | | | | | | | | | | |
| Syndromic screening and syndromic management for STIs | B | |  | |  |  | Quarterly in both participants until conception occurs | | | | | | | | | | | |
| Collect blood specimen | B | | B | | B | B | Monthly in all participants until conception occurs, then quarterly thereafter, and at last study visit | | | | | | | | | | | |
| Collect urine for pregnancy testing | A♀ | | A♀ | | A♀ | A♀ | Monthly in all women until conception occurs | | | | | | | | | | | |
| Collect urine for urinalysis for proteinuria | HIV+ | | If indicated by symptoms (per Zimbabwean NMTPAC/MOHCC guidelines) | | | | | | | | | | | | | | | |
| Collect vaginal swab for prostate specific antigen (PSA) testing |  | | A♀ | | A♀ | A♀ | Monthly in all women until conception occurs, then quarterly thereafter, and at last study visit | | | | | | | | | | | |
| Provide folic acid supplementation |  | | A♀ | | In all women and through the end of pregnancy | | | | | | | | | | | | | |
| Collect hair sample |  | |  | Monthly in participants using ART or PrEP until conception occurs, quarterly thereafter in participants using ART or PrEP, and at last study visit | | | | | | | | | | | | | | |
| Provide ART |  | | Monthly for HIV+ who opt for ART through end of pregnancy | | | | | | | | | | | | | | | |
| Provide PrEP |  | | Monthly for HIV- who opt for PrEP through conception; counsel and re-consent at conception for PrEP use through end of pregnancy | | | | | | | | | | | | | | | |
| Provide artificial vaginal insemination materials |  | |  | |  |  | Monthly for couples who opt for artificial vaginal insemination until conception | | | | | | | | | | | |
| Collect semen sample for semen washing and intrauterine insemination IUI |  | |  | |  |  | Monthly for couples who opt for semen washing with IUI until conception | | | | | | | | | | | |
| Provide condoms | B | | For all participants at each visit through study exit | | | | | | | | | | | | | | | |

| **Laboratory Procedures** | |  | **2 month run-in** | | | | **Up to 12 months of pregnancy attempts and follow-up** | | | | | | | | | | | | | | | | | | | | |
| --- | --- | --- | --- | --- | --- | --- | --- | --- | --- | --- | --- | --- | --- | --- | --- | --- | --- | --- | --- | --- | --- | --- | --- | --- | --- | --- | --- |
| **Local laboratory in Harare & Chitungwiza** | **Screening** | | **Enrollment M0** | | **M**  **1** | **M**  **2** | **M**  **3** | **M**  **4** | **M**  **5** | | **M**  **6** | | **M**  **7** | **M**  **8** | | **M**  **9** | | **M**  **10** | | **M**  **11** | | **M**  **12** | | **M**  **13** | | **M**  **14** | |
| Hemoglobin testing | B | |  | |  |  |  |  |  | |  | |  |  | |  | |  | |  | |  | |  | |  | |
| Visual inspection with acetic acid (VIA), lugol's iodine (VILI) | A♀ | |  | |  |  |  |  |  | |  | |  |  | |  | |  | |  | |  | |  | |  | |
| HBV antigen testing | B | |  | |  |  |  |  |  | |  | |  |  | |  | |  | |  | |  | |  | |  | |
| Syphilis RPR testing |  | | B | |  |  |  |  |  | |  | |  |  | |  | |  | |  | |  | |  | |  | |
| Serum creatinine (and estimated creatinine clearance) | B | | per NMTPAC/MOHCC guidelines | | | | | | | | | | | | | | | | | | | | | | | | |
| CBC | HIV+ | | per NMTPAC/MOHCC guidelines | | | | | | | | | | | | | | | | | | | | | | | | |
| Dipstick urinalysis for proteinuria | HIV+ | | per NMTPAC/MOHCC guidelines | | | | | | | | | | | | | | | | | | | | | | | | |
| CD4 count | HIV+ | | per NMTPAC/MOHCC guidelines | | | | | | | | | | | | | | | | | | | | | | | | |
| HIV antibody | B | | HIV- | | HIV- | HIV- | Monthly in the HIV- partner until conception occurs, quarterly thereafter, and at last study visit | | | | | | | | | | | | | | | | | | | | |
| HIV viral load |  | | HIV+ | |  | HIV+ | Monthly in the HIV+ partner until conception occurs, quarterly thereafter, and at last study visit | | | | | | | | | | | | | | | | | | | | |
| Urine pregnancy testing | A♀ | | A♀ | | A♀ | A♀ | Monthly in all women until conception occurs | | | | | | | | | | | | | | | | | | | | |
| Prostate specific antigen (PSA) testing |  | | A♀ | | A♀ | A♀ | Monthly in A♀ until conception occurs, then quarterly thereafter, and at last study visit | | | | | | | | | | | | | | | | | | | | |
| **Laboratory at UCSF** | **Screening** | | **Enrollment M0** | | **M**  **1** | **M**  **2** | **M**  **3** | **M**  **4** | | **M**  **5** | | **M**  **6** | **M**  **7** | | **M**  **8** | | **M**  **9** | | **M**  **10** | | **M**  **11** | | **M**  **11** | | **M**  **13** | | **M**  **14** |
| HIV genotype |  | | SC - This testing will occur at any point there is an HIV sero-conversion | | | | | | | | | | | | | | | | | | | | | | | | |
| Hair sample adherence testing |  | |  | Monthly among those using ART or PrEP until conception occurs, quarterly thereafter, and at study exit | | | | | | | | | | | | | | | | | | | | | | | |

KEY: M– study month; B – Both participants in the couple; A♀– ALL (HIV+/HIV-) women; HIV+ – HIV-infected partner; HIV- – HIV-negative partner; ART– HIV-infected participants who select ART as a strategy; PrEP– HIV-negative participants who select PrEP as a strategy; VI – couples who select VI as a strategy; SC – partners who seroconvert and the corresponding HIV-infected partner; *this testing will occur at any point there is a seroconversion.¥ Participants who are found to have <95% adherence on the three-day self-report questionnaire will be referred for adherence counseling

Appendix B. Schedule of Events in Pregnancy and Postpartum

Once female participants become pregnant, they and their partners will continue to have quarterly visits through 3 months post-partum.

|  | Phase/Study Month | | | | |
| --- | --- | --- | --- | --- | --- |
| **Procedure or Evaluation** | **Pregnancy** | | | **Post-Partum** | |
| **Clinical Procedures** | M 3 | M 6 | M 9 | 6 weeks | 12 weeks |
| **Study visit for couple** | B | B | B | B | B |
| Collect/update locator information | B | B | B |  |  |
| Targeted Clinical Questionnaire & AEs | B | B | B |  |  |
| Questionnaire on Patient Satisfaction with Safer Conception Method | Quarterly in all participants, and at last study visit | | | | |
| Adherence Questionnaire on Safer Conception Strategies, Sexual Behavior, and Condom Use | Quarterly in all participants, and at last study visit | | | | |
| Questionnaire on Beliefs About Methods of Safer Conception, Relationship Power, and HIV-related Stigma | At last study visit | | | | |
| Abstract data from Antenatal Care (ANC) card | A♀ | A♀ | A♀ | A♀ | A♀ |
| Provide condoms | B | B | B | B | B |
| Collect blood specimen | B | B | B | B | B |
| Collect vaginal swab for prostate specific antigen (PSA) testing | A♀ | A♀ | A♀ |  |  |
| Urine sample collection | HIV+ (per NMTPAC/MOHCC guidelines) | | | | |
| Hair sample collection | ART or PrEP | | |  | ART or PrEP* |
| **Study visit for Infant** |  |  |  | HEI | HEI |
| Collection blood specimen |  |  |  | HEI | HEI |
| Abstract data from HIV Exposed Infant (HEI) Follow Up Card |  |  |  | HEI | HEI |
| **Laboratory Procedures** |  |  |  |  |  |
| Local laboratory in Harare & Chitungwiza | M 3 | M 6 | M 9 | 6 weeks | 12 weeks |
| Serum creatinine (and estimated creatinine clearance) | HIV+ (per NMTPAC/MOHCC guidelines) | | |  |  |
| CBC | HIV+ (per NMTPAC/MOHCC guidelines) | | |  |  |
| Dipstick urinalysis for proteinuria | HIV+ (per NMTPAC/MOHCC guidelines) | | |  |  |
| CD4 count | HIV+ (per NMTPAC/MOHCC guidelines) | | |  |  |
| HIV antibody | HIV- | HIV- | HIV- | HIV- | HIV- |
| HIV viral load | HIV+ | HIV+ | HIV+ | HIV+ | HIV+ |
| Prostate specific antigen (PSA) testing | A♀ | A♀ | A♀ |  |  |
| Infant HIV DNA PCR |  |  |  | HEI | HEI |
| Infant Hemoglobin |  |  |  | HEI |  |
| **Laboratory at UCSF** |  |  |  |  |  |
| HIV genotype | At any visit there is an HIV seroconversion | | | | |
| Hair sample adherence testing | Quarterly among those using ART or PrEP | | | | |

KEY: M– study month; A♀ – all female partners; HEI– HIV-exposed infants; HIV+ – HIV-infected partner; HIV- – HIV-negative partner; ART– HIV-infected participants who select ART as a strategy; PrEP– HIV-negative participants who select PrEP as a strategy; *PrEP will be discontinued among breastfeeding women, no hair sample will be taken

APPENDIX C: Anticipated Timeline

| **Activity** | **Jan-Sept 2016** | **Oct –Dec**  **2016** | **Jan -Dec 2017** | **Jan – May 2018** |
| --- | --- | --- | --- | --- |
| Planning & Development: | x |  |  |  |
| - Develop study questionnaires, consents, and standard operating procedures |  |  |  |  |
| - Employ and train study staff  - IRB approvals |  |  |  |  |
| Objective 1: Pilot study |  |  |  |  |
| - Screening/Enrollment |  | x |  |  |
| - Safer conception follow-up |  | x | x |  |
| - Pregnancy/birth/infertility follow-up |  |  | x | x |
| Objective 2: IDIs |  |  |  | x |
| Objective 3: Costing study |  | x | x | x |
| Analysis and manuscript preparation |  |  |  | x |
