# Supplementary material for: Preventing HIV and achieving pregnancy among HIV sero-different couples: Pilot study of a safer conception intervention in Zimbabwe
Source: PLOS Glob Public Health. 2023 Feb 24;3(2):e0000796. doi: 10.1371/journal.pgph.0000796 (PMC10022125; doi:10.1371/journal.pgph.0000796)
Supplement: S2 Text — (DOCX) [file pgph.0000796.s004.docx]

**S2 Text - SAE Listing**

**SAE #1** - First-term miscarriage. Female participant (HIV-uninfected, 32 years, parity 3) chose PrEP (TDF-FTC) and her partner (HIV-infected, 52 years) chose ART with viral load monitoring as safer conception strategies. She fell pregnant during follow-up and opted to continue PrEP (TDF-FTC) after conception.  She returned for a regular quarterly follow-up visit and reported that she had a miscarriage. Her estimated gestational age at the time of miscarriage was approximately 8 weeks based on last menstrual period. She decided against seeking clinical care when her symptoms arose. On examination at the clinic she was stable with normal vitals and had no mucosal pallor. Pelvic examination was normal- not bleeding and cervical os was closed. The medical officer made a diagnosis of a complete miscarriage. Patient was referred for supportive counselling. The event was considered resolved, and this couple continued in the study, per protocol. This AE was classified as ‘unlikely to be related’ to PrEP (TDF-FTC) because there is no direct evidence that PrEP (TDF-FTC) contributes to miscarriages, but there also is not definitive evidence that it is 100% non-contributing. The participant also noted at this visit that prior to joining the study she had experienced a previous first trimester miscarriage at 12 weeks. No other known risk factors or precipitating factors (such as trauma, fever/severe illness) were present.

**SAE #2** – Pelvic Inflammatory Disease. Female participant (HIV-uninfected, 34 years, parity 3) presented for screening into the study. Upon screening examination, there was an offensive per vaginal discharge. She and her partner were managed and treated as per national STI guidelines. There was improvement following the antibiotics course. She was enrolled into the study and chose PrEP (TDF-FTC) as her safer conception strategy (her partner chose ART with viral load monitoring). Two days after enrollment, she presented at our clinic with a day’s history of pelvic pain associated with fever, palpitations and shortness of breath. She was pyrexic, tachycardic, and hypotensive. Pelvic examination revealed a bloody offensive discharge, cervical excitatory tenderness and adnexal tenderness. Pelvic ultrasound was done and the medical officer made a diagnosis of Pelvic Inflammatory Disease (PID) to rule out pelvic abscess. She was given stat doses of intravenous antibiotics and intravenous fluids at the study clinic then referred to the Chitungwiza Central hospital where she was admitted. This event was classified as ‘unrelated’ to PrEP because it was a worsening of a baseline condition.  She fully recovered and was discharged 4 days later. She and her partner continued to participate in the study.

**SAE #3** – First-term miscarriage.  Female participant (HIV-uninfected, 29 years, parity 2) chose PrEP (TDF-FTC) and her male partner (HIV-infected, 36 years) chose ART with viral load monitoring as safer conception strategies. She fell pregnant during follow-up and opted to continue PrEP (TDF-FTC) after conception.  She called the study coordinator with reports of bleeding and cramping, and was referred to her local clinic for immediate clinical evaluation. She was subsequently referred to Parirenyatwa hospital for further assessment. Ultrasound scan confirmed an incomplete miscarriage and she had also developed severe anaemia from the bleeding. She was admitted to hospital, had manual vacuum aspiration done, was given 2 units of blood, and was provided with treatment. She was discharged in stable condition after 3 days.  Her estimated gestational age at the time of miscarriage was approximately 11 weeks based on last menstrual period. This AE was classified as ‘unlikely to be related’ to PrEP (TDF-FTC) because there is no direct evidence that PrEP (TDF-FTC) contributes to miscarriages, but there also is not definitive evidence that it is 100% non-contributing. No other known risk factors or precipitating factors (such as trauma, fever/severe illness) were present. She and her partner continued to participate in the study. (Note: Six months later, she and her partner conceived during the study and gave birth to a healthy full term baby. Both mother and baby were HIV-uninfected at study exit).

**SAE #4** – First-term miscarriage.  Female participant (HIV-uninfected, 33 years, parity 2, history of pregnancy induced hypertension) and her male partner (HIV-infected, 34 years old) chose ART with viral load monitoring, as well as semen washing with intrauterine insemination as safer conception strategies. She fell pregnant during follow-up. At 12 weeks gestations as estimated from last menstrual period, she called the study staff to inform that she had per vaginal bleeding with clots, went to a local clinic where she was referred to Harare Central Hospital for further management. She was attended at Casualty department where she had evacuation done for retained products of conception due to incomplete miscarriage. Vital observations were checked at hospital and her blood pressure was 94/50mmHg, pulse 82b/m and temperature 36.2C. She was given intravenous fluids of normal saline 1 litre to run fast. Blood was collected for urgent full blood count and result was 11.3g/dl. Participant was discharged the same day after stabilization. She was discharged on Metronidazole 400mgs tds p.o and Amoxicillin 500mgs tds p.o both drugs to be taken for 7 days. She was reviewed at the study clinic two weeks later, and had fully recovered. This adverse event was classified as ‘unrelated’ to study participation or drugs because the participant was not taking ART or PrEP. She and her partner continued to participate in the study. (Note: Five months later, she and her partner conceived during the study and gave birth to a healthy full-term baby. Both mother and baby were HIV-uninfected at study exit).

**SAE #5** – Hospitalization to rule out possible Typhoid. Male participant (HIV-uninfected, 34 years) was enrolled into the study with his wife (HIV-infected). She chose ART with viral load monitoring and he chose PrEP as safer conception strategies. When she fell pregnant, he opted to discontinue PrEP. Three weeks after she delivered a full-term baby (HIV-uninfected), the male partner was admitted to Chitungwiza Hospital with a history of headache, vomiting, chills and fever, abdominal pain, and joint pain. They suspected typhoid. Participant was treated with intravenous antibiotics and analgesia (Rocephine 1g BD, Gentamycin 160mg od, Metronidazole 500mg iv stat, Paracetamol 1g tds). After recovering, he was discharged 4 days later in good condition. Rectal swab was negative. The couple continued to participate in study follow-up visits. This event is considered ‘unrelated’ to study participation or study strategies because the participant had not been using a safer conception method for previous 9 months.

**SAE #6** – Intrauterine death/Stillbirth. Female participant (HIV-infected, 32 years, parity 0, baseline CD4 count 845) and her partner (HIV-uninfected, 39 years) were enrolled into the study. Participant was taking ART for previous 21 months. The couple opted for ART and viral load monitoring, as well as PrEP (TDF-FTC) for the male partner as their methods of safer conception. During a regularly scheduled study visit, the participant tested positive for pregnancy by urine pregnancy test and was referred for antenatal care at the local clinic, as per protocol. Throughout study participation and pregnancy, the participant’s viral load remained undetectable, her CD4 count ranged between 845-861 cells/mm, she took iron and folic acid supplements, she had no signs or symptoms of STIs, and no reports of trauma. She returned to the study clinic for her regularly scheduled quarterly study visit during pregnancy (Month 3, Month 6, and Month 9), with no remarkable signs or symptoms, and reported she was attending her antenatal visits as recommended. Data from her local antenatal health card suggest she was not anaemic, no pre-eclampsia (normal blood pressure; under 130/90mmHg), no proteins or glucose detected in urine, no per vaginal bleeding, no vaginal sores, and no significant weight loss. Fetal growth as measured by fundal height was appropriate, and a normal fetal heart rate was detected, most recently at the 36-week antenatal visit. Both she and her partner had no signs or symptoms of sexually transmitted infections and tested negative for syphilis. After not perceiving fetal movement for 2 days, at 38 weeks, the participant presented at her local antenatal clinic experiencing mild labour contractions and complaining of backache. At the antenatal clinic, the fetal heart rate was not detected, and she was immediately referred to Chitungwiza Hospital for further management. The following day, the participant progressed with the stages of labour and delivered a macerated stillborn fetus (2700 grams) with normal gross phenotype. The placenta was showing signs of necrosis. According to hospital delivery notes, the clinical history and abrupt loss of fetal movements in this case suggest the most likely cause to be concealed placental hemorrhage. Fetal autopsy was not done. Oxytocin 10iu was given soon after the delivery. Later that day, after she stabilized, the participant was discharged home on Amoxyl 500mg tds and Metronidazole 400mg tds for 7 days and was reviewed at a local ANC clinic after 6 weeks. The couple was offered counselling by study staff, as well as referrals for additional bereavement counselling. This unfortunate event is considered ‘probably not related’ to study participation or study strategies because there is no direct evidence that HAART contributes to stillbirth, but there also is not definitive evidence that it is 100% non-contributing.
